# Microbial Community Changes in 26,500-Year-Old Thawing Permafrost

**DOI:** 10.3389/fmicb.2022.787146

**Published:** 2022-03-24

**Authors:** Maria Scheel, Athanasios Zervas, Carsten S. Jacobsen, Torben R. Christensen

**Affiliations:** ^1^Department of Ecoscience, Arctic Research Centre, Aarhus University, Roskilde, Denmark; ^2^Department of Environmental Science, Aarhus University, Roskilde, Denmark; ^3^Oulanka Research Station, Oulu University, Oulu, Finland

**Keywords:** permafrost erosion, abrupt thaw, 16S, fungi, Greenland, amplicon sequencing, soil microbiome, biodiversity

## Abstract

Northern permafrost soils store more than half of the global soil carbon. Frozen for at least two consecutive years, but often for millennia, permafrost temperatures have increased drastically in the last decades. The resulting thermal erosion leads not only to gradual thaw, resulting in an increase of seasonally thawing soil thickness, but also to abrupt thaw events, such as sudden collapses of the soil surface. These could affect 20% of the permafrost zone and half of its organic carbon, increasing accessibility for deeper rooting vegetation and microbial decomposition into greenhouse gases. Knowledge gaps include the impact of permafrost thaw on the soil microfauna as well as key taxa to change the microbial mineralization of ancient permafrost carbon stocks during erosion. Here, we present the first sequencing study of an abrupt permafrost erosion microbiome in Northeast Greenland, where a thermal erosion gully collapsed in the summer of 2018, leading to the thawing of 26,500-year-old permafrost material. We investigated which soil parameters (pH, soil carbon content, age and moisture, organic and mineral horizons, and permafrost layers) most significantly drove changes of taxonomic diversity and the abundance of soil microorganisms in two consecutive years of intense erosion. Sequencing of the prokaryotic 16S rRNA and fungal ITS2 gene regions at finely scaled depth increments revealed decreasing alpha diversity with depth, soil age, and pH. The most significant drivers of variation were found in the soil age, horizons, and permafrost layer for prokaryotic and fungal beta diversity. Permafrost was mainly dominated by Proteobacteria and Firmicutes, with *Polaromonas* identified as the most abundant taxon. Thawed permafrost samples indicated increased abundance of several copiotrophic phyla, such as Bacteroidia, suggesting alterations of carbon utilization pathways within eroding permafrost.

## Introduction

Ambient temperatures in the Arctic have increased by 3.1°C between 1971 and 2019, equaling roughly three times that of the global average temperature increase (AMAP, 2021). This trend is predicted to continue over the next 30 years with a local increase of as much as 3–6°C (IPCC, 2019). These complex consequences are projected to intensify in future scenarios (IPCC, 2021), including the recession of glacial and ice sheet extent, a decline in sea ice, and the thawing of permafrost (AMAP, 2021). These soils consist of a seasonally thawing active layer and the underlying permafrost table, which has remained permanently frozen for at least two consecutive years. Permafrost-affected soils take up a quarter of the global soil surface while storing more than half of the global soil carbon (Tarnocai et al., 2009; Schuur et al., 2015). The Northern hemisphere permafrost zone currently contains roughly twice the amount of carbon as the atmosphere is estimated to store (Schuur and Mack, 2018). These geological reservoirs are at risk as Arctic permafrost soils have warmed by 2–3°C since the 1970s and by 0.3°C between 2007 and 2016, recorded within the coldest permafrost sites, such as found in Greenland (IPCC, 2019; AMAP, 2021). An increase of 3.3–10°C above the 1985–2014 average temperature is expected by 2100 (AMAP, 2021). Thermally induced gradual active layer deepening makes formerly frozen carbon stocks available for both deeper rooting vegetation and microbial decomposition (Hayes et al., 2014; AMAP, 2019). In contrast, abrupt thaw, often triggered by melting ground ice, can impact deeper permafrost carbon by means of deep incisions into the soil profile. Currently, these processes affect up to 20% of all permafrost-affected areas, although by 2100, up to 60% could be impacted (Turetsky et al., 2020). A global increase of 1.5–2°C is projected to make 28–53% of all permafrost organic carbon bioavailable (IPCC, 2019). Turetsky et al. (2020) modeled that 613–802 and 624 Tg CO_2_ equivalents per year loss to the atmosphere could be accounted to gradual and abrupt permafrost thaw, respectively, until 2100 under the RCP8.5 scenario (IPCC, 2019). Permafrost carbon has accumulated over geological timescales and, once decomposed into gases, its degradation is an irreversible climate change indicator (AMAP, 2021).

When studying the connection between climate change and Arctic ecosystems (Cavicchioli et al., 2019), the importance of soil microorganisms is often underestimated. They are ecological sentinels of changing environmental conditions due to their fast growth rates and diverse metabolic potential (Edwards et al., 2020). In previous studies, DNA has been successfully extracted from up to 1 million-year-old permafrost (Willerslev et al., 2004) as well as 600,000-year-old DNA from viable permafrost organisms (Johnson et al., 2007). Microbial activity has been measured in permafrost samples as cold as −39°C (Panikov et al., 2006). Cryophilic organisms are adapted physiologically to sub-zero temperatures, but also to desiccation, high salinity, nutrient scarcity, and ground radiation, by adapting membrane fluidity, forming spores, and metabolizing complex organic sources (Collins and Margesin, 2019). As permafrost soils thaw, the existing cold-adapted microbial community, containing bacteria, archaea, viruses, and micro-eukaryotes such as fungi, is strongly affected by warmer temperatures, higher soil water content from melted ground ice, and increased nutrient availability from deeper rooting vegetation (Margesin, 2009). Not only do increasing ambient temperatures impose changing ecological conditions on the soil microbiome, but metabolic rates increase, and microorganisms can decompose diverse carbon substrates into climate-active gases (Jansson and Taş, 2014; Mackelprang et al., 2016; Schuur and Mack, 2018). As recently reviewed, microbial decomposition rates of ancient permafrost carbon are one aspect of the essential linkage between climate change and environmental microbiology and can greatly influence the carbon flux between permafrost and the atmosphere, as well as the linkage with the global nitrogen cycle (Cavicchioli et al., 2019).

Interest in Arctic permafrost has recently increased, rooted partly in the speed of irreversible erosion processes and knowledge gaps in dominant taxa and their metabolic potential on decomposing ancient permafrost matter during microbial succession in thawing permafrost soils (Edwards et al., 2020). Research in this area has been conducted but is still rare and focused on a few sites and mainly carried out using laboratory incubations and cultivation studies (Mackelprang et al., 2016; Metcalfe et al., 2018). In this paper, we investigated *in situ* which physicochemical soil parameters drive the microbial community composition and diversity changes with vertical abrupt thermal erosion as well as after 1 year of additional thawing. These insights will facilitate the development of quantitative approaches and builds a taxonomic baseline for future genetic research in this field, such as comparative metatranscriptomics. Furthermore, the rarely observed impact of abrupt loss of ancient microbiomes and the following ecological succession enhances our understanding of ecological cascades under climate change.

## Materials and Methods

### Sampling and Site Description

The Zackenberg Valley, Northeast Greenland (74°30′N, 20°30′W), is a wide lowland valley dominated by continuous permafrost. According to Elberling et al. (2010), annual air temperatures averaged −9.5°C between 1996 and 2007 and summer temperatures varied between 3 and 7°C from 1996 to 2007 (Hansen et al., 2008; Hollesen et al., 2011). The vegetation in the valley consists primarily of wet hummocky fens and low shrub and graminoid species (Elberling et al., 2008), while the lowlands east of the Zackenberg river are *Cassiope tetragona* and grass heathland (Bay, 1998). Permafrost up to 1 m deep had an average temperature of −2°C in summer and −14°C in winter between 1997 and 2006 (Christiansen et al., 2008). The average maximum active layer thickness ranged from 40 cm up to 2 m in depth and increased by 0.8–1.5 cm per year between 1996 and 2012 (Elberling et al., 2013). Long-term soil temperature data from 1995 to 2020 from the monitoring site SAL-1 at depths of 0, 20, 40, 60, 80, and 100 cm were retrieved from the Greenland Ecosystem Monitoring database^1^ ^,2^ and averaged for August and September.

After an intense snowmelt event in the 2018 summer season, a formerly existing minor thermokarst, a depression of the eroding permafrost soil surface, collapsed 400 m north-east of the Zackenberg Research Station. The incision into the soil profile was about 1 m deep and created an erosion gully that led toward the Zackenberg River. An ice lens at 40–60 cm depth was visible, which had melted in 2019 before sampling took place (Supplementary Figure 1). The thaw depth was measured with a thin steel pole pushed into the soil vertically three times until resistance indicated the frozen permafrost table had been reached (Christiansen, 1999). Based on these measurements, the layers were defined as the active layer (the long-term seasonally thawing top 40 cm), the transition zone of newly thawed permafrost material (40–70 cm in 2019 and 40–90 cm in 2020), and permafrost as the continuously frozen layer below these (below 70 cm in 2019 and 90 cm in 2020) as visualized in Supplementary Figure 2. In 2019 and 2020, three biological replicates were taken aseptically for every 10 cm interval until a depth of 90 or 100 cm, respectively. Due to the long transport chain without stable frozen transport technology and the bias, inconsistent freeze-thawing processes were shown to inflict on cold-adapted microbiomes (Lim et al., 2020), all samples were stored at 4°C at the Research Station before controlled, cooled transportation to Denmark, then stored at 4°C until further laboratory analysis took place as reported earlier (Gittel et al., 2014b).

### Loss on Ignition and Radiocarbon Dating

For radiocarbon dating, field-wet soil per 10-cm horizon for 2019 was sifted in technical triplicated with a 0.5-mm sieve in deionized water to retrieve macro plant residues for further analysis, while the roots were removed under a stereomicroscope. To counter the low biomass, the triplicates were pooled per depth, before chemical pre-treatment with HCl and NaOH. The samples were graphitized before measuring the ^14^C isotope activity using an accelerator mass spectrometer (Radiocarbon Dating Laboratory, Lund University, Lund, Sweden). The obtained peaks were compared to established ^14^C calibration curves, considering anthropogenic atmospheric ^14^C activity changes. The results were reported as age in ^14^C years in BP (before present = AD 1950) from 1650 to 1950 using the terrestrial calibration curve IntCal13 (Reimer et al., 2013) and for younger datings than 1963 in fM (fraction modern) using the Levin post-Bomb calibration (Levin and Kromer, 2004).

Loss on ignition was performed in technical triplicate for each 10-cm depth interval per year on wet soil, by air-drying at 70°C for 48 h, then weighing to determine the weight-based relative soil water (H_2_O) loss. The samples were burned at 450°C for 2 h in ceramic cups and weighed to obtain the weight-based relative organic carbon content (SOM). To measure the pH, 10 ml of air-dried soil in triplicates were added to 50 ml of 1 M KCl in Falcon Tubes in technical triplicate. After shaking for 1 h and resting for 1.5 h, the pH was measured with a Mettler Toledo FiveEasy Plus™ pH Meter (Mettler Toledo GmbH, Gieβen, Germany).

Based on the radiocarbon age and organic matter content of the soil, we defined each a surface (1) and buried (2) organic (O) and mineral (M) horizon, resulting in two organic (O1, O2) and mineral (M1, M2) horizons (Table 1 and Supplementary Figure 2). In contrast, layers were defined based on the thermal state of the soil with a local active layer (AL) designated as the seasonally thawing material until 40 cm. The transition zone (TZ) was designated as former permafrost material which had thawed since the collapse to 70 cm in 2019 and 90 cm in 2020. The underlying permafrost layer (PF) remained frozen, indicated by visible ice crystals in the material (Supplementary Figure 3).

**TABLE 1:** Physicochemical soil parameters were measured at the sampling site and included radiocarbon dating (^14^C), which was measured only in 2019 and was calculated in fM (*) for samples younger than 1963 or BP (**) for samples older than 1945.

Depth [cm]	^14^C [*fM **BP]	H_2_O [%]		SOM [%]		pH		Horizon	Layer	
				
		2019	2020	2019	2020	2019	2020		2019	2020
0–10	*1.04	7.94 ± 1.03	28.80 ± 3.38	16.01 ± 4.22	8.73 ± 1.82	3.87 ± 0	4.22 ± 0.03	O1	AL	
	
10–20	*1.13	11.77 ± 0.93	22.52 ± 0.73	7.09 ± 1.71	5.39 ± 0.83	4.46 ± 0.05	4.02 ± 0.05	M1		
20–30	*1.16	11.83 ± 0.34	22.05 ± 3.30	5.75 ± 0.21	10.19 ± 3.22	4.25 ± 0.02	4.29 ± 0.01			
30–40	*1.20	13.13 ± 3.12	26.57 ± 0.83	5.85 ± 2.23	13.68 ± 0.44	4.73 ± 0.09	4.25 ± 0.01			

40–50	**2,635	28.27 ± 4.92	7.73 ± 1.58	24.42 ± 4.78	2.59 ± 0.68	5.08 ± 0.05	4.63 ± 0.03	O2	TZ	TZ
50–60	**3,770	23.49 ± 1.92	15.61 ± 6.28	15.76 ± 4.50	2.93 ± 0.30	5.45 ± 0.10	4.13 ± 0.02			
	
60–70	**26,500	14.14 ± 8.43	8.72 ± 2.53	3.78 ± 0.09	1.52 ± 0.16	6.54 ± 0.05	4.86 ± 0.05	M2		
70–80	**22,100	16.31 ± 1.09	6.35 ± 0.60	3.99 ± 0.25	1.10 ± 0.24	7.01 ± 0.01	4.48 ± 0		PF	
80–90	**26,200	1.65 ± 4.43	7.96 ± 0.61	1.78 ± 0.30	1.00 ± 0.20	6.61 ± 0	4.57 ± 0.01			
90–100	NA	NA	7.80 ± 0.59	NA	1.04 ± 0.26	NA	4.91 ± 0.01			PF

*In both years, weight-based relative soil moisture (H_2_O) and soil organic matter (SOM) were measured based on loss on ignition and the pH was determined in triplicates and standard deviation is indicated (±). Layers included the active layer (AL, 0-40 cm) as long-term seasonally thawing material above the former ice lens, the transition zone (TZ, 2019: 40-70 cm, 2020: 40-90 cm) as the newly thawed material since the collapse, and permafrost (PF, 2019: > 70 cm, 2020: > 90 cm) as the still frozen ground. Based on SOM and ^14^C, four separate horizons were visible, namely the recent surface organic horizon (O1, 0-10 cm), the recent underlying mineral horizon (M1, 10-40 cm), the older buried organic horizon (O2, 40-60 cm), and an ancient mineral horizon (M2, > 60 cm).*

### DNA Extraction and Sequencing

DNA was extracted from 2 g field-wet soil per year and 10-cm depth interval for each biological replicate no later than 67 days after sampling, using the DNAeasy^®^ PowerSoil^®^ Kit (Qiagen, Hilden, Germany) according to the manufacturer’s instructions. Given the low biomass and DNA concentration, these extraction triplicates were pooled for sequencing in order to maximize coverage. The resulting extracts were quantified with a Qubit^®^ 2.0 Fluorometer (Thermo Fisher Scientific, Life Technologies, Roskilde, Denmark). For prokaryotic DNA, the 16S rRNA V3-V4 region was amplified with the primer set F341 (5′-CCTAYGGGRBGCASCAG-3′) and R806 (5′-GGACTACNNGGGTATCTAAT-3′) (Takahashi et al., 2014), while for fungal sequences the ITS2 region was targeted with the primers ITS1F2 (5′-GAACCWGCGGARGGATCA-3′) and ITS2 (5′-GCTGCGTTCTTCATCGATGC-3′) (Gaylarde et al., 2017). For the PCR, negative controls confirmed absence of contamination. The final PCR products were cleaned with magnetic beads (MagBio Genomics Inc., Gaithersburg, Maryland, US) and fresh 80% ethanol, and stored in 1x TE buffer at −20°C. The quality and size of the amplicons were verified by running an agarose gel and screening with Tapestation 4150 (Agilent Technologies, Santa Clara, California, US). Paired Illumina MiSeq 2 × 300 bp sequencing was performed at Teknologisk Institut, Taastrup, Denmark.

### Community and Diversity Analysis

All of the following analyses were performed using the QIIME 2 pipeline and plugins version 2019.10.0 (Bolyen et al., 2019). Raw sequence reads were filtered by removing the multiplexing and gene region primer sequences with Cutadapt (Martin, 2011). The 50–60 cm 16S samples from 2020 and 0–10 cm and 60–70 cm ITS sequences from 2019 were removed due to an insufficient quality of reads after filtering. Noisy and redundant sequences were removed, to reduce sequencing errors and chimeras with dada2 denoise (Callahan et al., 2017). Unique filtered sequences were defined as amplicon sequencing variants (ASVs) and used for downstream analysis (Supplementary Table 1).

The prokaryotic ASVs were classified with the closest taxonomic affiliation with the classifier sklearn (Pedregosa et al., 2011) against the SILVA database (version 138_99) and the UNITE database version 8.2 (version 2020-02-20, Quast et al., 2013) with global and 97% singletons for fungi (Nilsson et al., 2019). The phylogeny was created with fasttree on classified sequences (Version 2.1.10, Price et al., 2010). For each sample, alpha phylogenetic diversity (PD) was determined with Faith’s PD index. Several other diversity indices were calculated but not used for downstream analysis (Supplementary Table 1). Beta diversity, as the variation of diversity across several samples, was calculated as Bray–Curtis dissimilarities (BC) as a non-phylogenetic index, and weighted Unifrac as pairwise phylogenetic distances.

### Statistical Significance Tests

On ASV level, phylogenetic alpha diversity correlation was tested with environmental metadata for variation, including pairwise Kruskal–Wallis covariation (non-parametric Mann–Whitney *U*-test) in QIIME2. Permutational multivariate analysis of variance (PERMANOVA) on 999 permutations was performed using the “adonis” function to determine significant drivers of community composition. A Principal coordinate analysis (PCoA) was performed on the weighted Unifrac distances and plotted as biplots, utilizing the Emperor QIIME2 plugin. Additional analyses were conducted using the “vegan” packages (Oksanen et al., 2019) in the R studio environment and R version 4.1.2 (R Core Team, 2021; R Studio Team, 2021). Correlation-based indicator species analysis (Dufrêne and Legendre, 1997) was performed on phyla abundances for each categorical parameter using the “indval” function. Non-Metric Multidimensional Scaling (NMDS) biplots with fitted environmental parameters were performed on BC dissimilarities based on the relative abundance of phyla.

## Results

### Physicochemical Soil Analyses

At our study site, the weight-based relative soil organic matter (SOM) content indicated a mainly organic top 10 cm horizon (O1). It was underlain by the silt and clay mineral horizon (M1), which reached until the active layer limit at 30–40 cm depth with an average SOM content < 10%. Both were dated to be younger than 58 years old. Formerly separated from these by ground ice, the 20-cm deep organic horizon (O2) from 50–60 cm depth indicated up to 3,841 years old underlying material and after the melting of the ice lens in 2019 highest relative, weight-based soil water content (H_2_O) of 28.27%. The deepest horizon (M2) from 60 cm depth and deeper consisted of sand and pebbles and up to 26,571-year-old material. This horizon included the upper permafrost table at 70 cm in 2019 and 90 cm in 2020. In 2019, the pH ranged from 3.87 of the top 10 cm to 7.01 at 70–80 cm depth, while the pH range in 2020 was stable throughout the profile from 4.01 to 4.91 (Table 1).

### Prokaryotic Community Composition and Abundance

**FIGURE 1:**
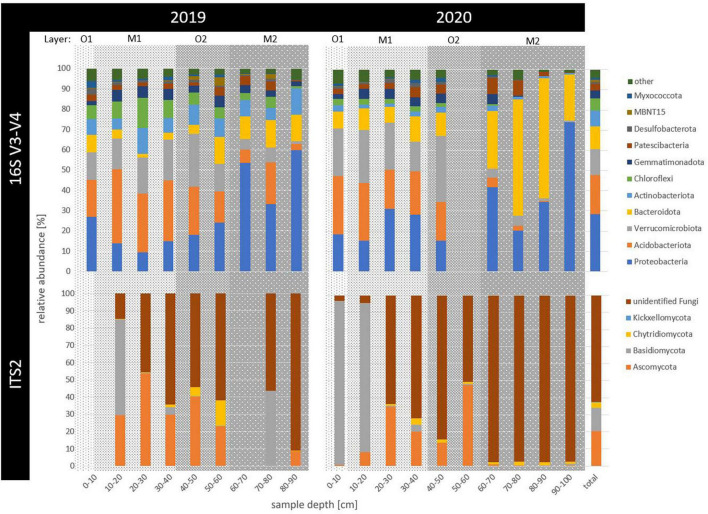
Relative abundance of prokaryotic (16S V3-V4) and fungal (ITS2) taxonomic phyla across 2 years of sampling (2019 and 2020) and in up to 100 cm deep permafrost (sample depth in cm). Phyla with less than 1% of relative abundance, including archaeal counts, were summarized as “other.” Horizons were indicated as gray background with the surface organic (O1) in light gray, buried mineral (M1) in medium gray, buried organic (O2) in darker and deepest, ancient mineral horizon (M2) in the darkest gray shading.

**FIGURE 2:**
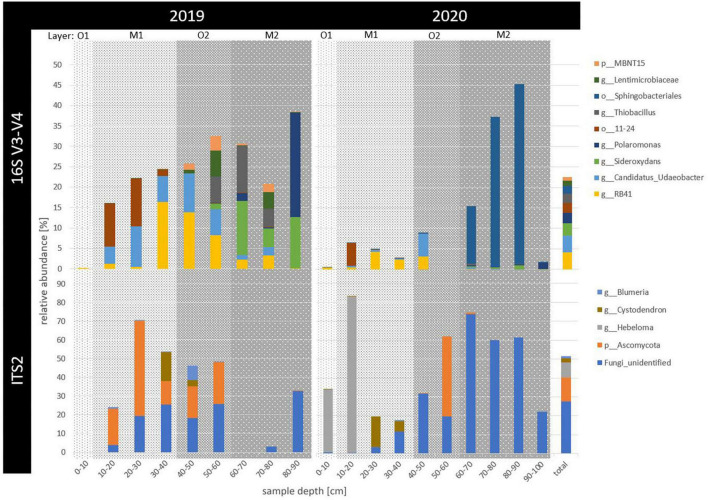
Relative abundance of the most abundant prokaryotic (16S V3-V4) and fungal (ITS2) ASVs across 2 years of sampling (2019 and 2020) and in up to 100 cm deep permafrost (sample depth in cm). Only ASVs with more than 1% relative abundance across all samples, including archaeal counts, were summarized to the highest taxonomic level possible. That taxonomic level is indicated as phylum (p_), order (o_), or genus (g_). Horizons were indicated as gray background with the surface organic (O1) in light gray, buried mineral (M1) in medium gray, buried organic (O2) in darker and deepest, ancient mineral horizon (M2) in the darkest gray shading.

Amplicon sequencing revealed a frequency of 10,256,910 counts across 18 samples for raw 16S sequences. After filtering, 25,471 unique reads were defined as prokaryotic amplicon sequence variants (ASVs, for sequencing statistics see Supplementary Table 1). Overall, four ASVs remained taxonomically unclassified and 75 ASVs were archaeal (0.19% relative abundance). Ubiquitous phyla included Proteobacteria (29%), Acidobacteriota (19%), Verrucomicrobiota (13%), Bacteroidota (11%), Actinobacteriota (8%), Chloroflexi (6%), Gemmatimonadota (4%), and Patescibacteria (3%), as well as rarer taxa such as Firmicutes (0.7%), Bdellovibrionata (0.3%), and Cyanobacteria (0.1%), relative abundance across all samples indicated in brackets (Figure 1). While no single ASV was shared by all samples, the 11 most abundant ASVs with overall > 1% of all counts depicted 20% of total relative abundance (Figure 2). The overall most dominant identified genera belonged to the order Burkholderiales (Comamonadaceae family: *Polaromonas* and *Rhodoferax*, Gallionellaceae family: *Sideroxydans*, and Hydrogenophilaceae family: *Thiobacillus*), while Chthoniobacterales were dominated by *Candidatus Udaeobacter*, and Pyrinomonadales by *RB41*.

Specifically in permafrost samples (2019: 70–90 cm, 2020: 90–100 cm), Proteobacteria increased with depth, with the order Burkholderiales accounting for up to 60% of reads in the permafrost samples. Within this order, the genus *Polaromonas* was the most abundant ASV in the deepest permafrost sample of 2019 (80–90 cm), where also Firmicutes mainly occurred. *Rhodoferax* had the highest counts in the deepest and only permafrost sample of 2020 (90–100 cm). *Sideroxydans* occurred most in permafrost samples in 2019 (60–90 cm). In 2019, *Thiobacillus* dominated the community most between 60 and 80 cm (Figure 2). Actinobacteriota abundance increased with depth but decreased from 2019 to 2020.

Within the freshly thawing permafrost material of the transition zone (2019: 40–70 cm, 2020: 40–90 cm), relative abundance of Bacteroidota increased between 2019 and 2020, driven mainly by the Sphingobacteriales order in 60–90 cm deep samples. The phyla MBNT15 and Patescibacteria also increased in the transition zone samples of 40–80 cm in 2019 and 60–80 cm in 2020, respectively. Verrucomicrobiota were present throughout the thawed samples of active layer and transition zone. In both years, the genus *Cand. Udaeobacter* was found both in the thawed mineral top and buried organic horizons until 60 cm depth (M1 and O2). Here, Acidobacteriota were also abundant and most represented by the orders 11–24 and Pyrinomonadales, which were most abundant between 10 and 50 cm depth, decreasing toward 2020. For the latter order, the genus *RB41* was among the highest counting ASVs in thawed soils in 2019, only seconded by *Cand. Udaeobacter*. Similarly, Chloroflexi also decreased in relative abundance with depth and time. Gemmatimonadota, Desulfobacterota, and Myxococcota abundance was rather constant throughout the samples, while the latter two had their maximal abundance in the surface organic horizon.

The indval analysis indicated the correlation of phyla with categorical environmental parameters (Supplementary Figure 6). Significantly more taxa were dominant in 2019, and only a few increased toward 2020, including Bacteroidota, Bdellovibrionota, and Fibrobacterota. These also correlated with increased abundance in the transition zone, together with MBNT15 and Patescibacteria, compared to other layers. While most taxa dominated the active layer (AL), Firmicutes indicated the strongest signal for significant abundance in permafrost samples. In contrast, each horizon had numerous relatively abundant taxa, without clearly outstanding trends (although Fibrobacterota and Firmicutes had their highest values in the deepest mineral horizon compared to other horizons; Supplementary Figure 6).

Archaeal relative abundance was marginal and even absent in permafrost samples. Phyla accounting for at least 1% of all archaeal counts included Crenarchaeota (59%), Nanoarchaeota (25%), Halobacterota (9%), Euryarchaeota (5%), and Thermoplasmatota (2% relative abundance within only archaeal ASVs across all samples). The two highest counting archaeal ASVs were assigned as Bathyarchaeia on 39% of all archaeal counts. The phyla Nanoarchaeota, Euryarchaeota, and Thermoplasmata each were driven mainly by one order, Woesearchaeales (25%), and the methanogenic Methanobacteriales (5%) and Methanomassiliicoccales (2%), respectively. Most of the Halobacterota abundance was also driven by other methanogenic taxa, such as the orders Methanomicrobiales and Methanosarcinales (7 and 2% total relative abundance).

### Fungal Community Composition and Abundance

Across 17 samples, raw ITS2 sequences had 1,543,256 counts, revealing 1,624 fungal ASVs after filtering, of which 63% remained unclassified. Only four phyla were classified as Ascomycota (21%), Basidiomycota (14%), Chytridiomycota (3%), and Kickxellomycota (< 0.005% relative abundance across all samples). No phylum appeared across all samples (Figure 1). For Basidiomycota, the genus *Hebeloma* was the most abundant in the first 20 cm in both years, accounting for 83% of the counts in the 10–20 cm deep sample in 2020 and 9% of all fungal counts. Ascomycota were particularly dominated between 0 and 50 cm depth by the genera *Cystodendron*, *Blumeria*, and *Neobulgaria*, accounting for 7% of all fungal counts (Figure 2). Most fungi within the permafrost samples remained unclassified.

### Microbial Diversity Along a Spatiotemporal Gradient

**FIGURE 3:**
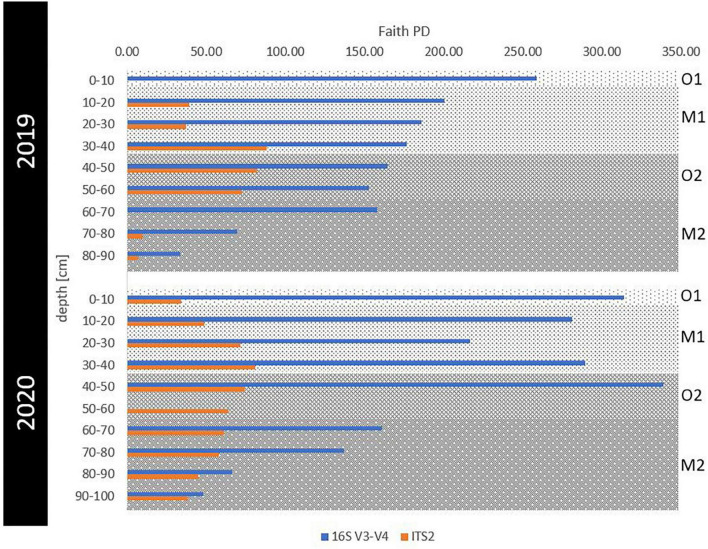
Phylogenetic alpha diversity for prokaryotic and fungal communities in eroding permafrost samples. The Faith’s PD index was visualized for 16S (blue) and ITS (orange) sequences per 10-cm depth intervals for 2019 and 2020. Horizons were indicated as gray background with the surface organic (O1) in light gray, buried mineral (M1) in medium gray, buried organic (O2) in darker and deepest, ancient mineral horizon (M2) in the darkest gray shading.

Sequence statistics and alpha diversity indices were calculated and are summarized in Supplementary Table 1. As all alpha diversity indices indicated similar trends, we utilized Faith’s PD for all downstream analyses. The prokaryotic alpha diversity (overall Faith’s PD = 181 ± 91) consistently decreased with depth in 2019, while in 2020, very high diversity was maintained throughout the whole active layer and peaked in 40–50 cm deep samples before decreasing with depth (Figure 3). In contrast, fungal phylogenetic alpha diversity was comparatively lower (overall Faith’s PD = 54 ± 24), peaking in both years at 30–40 cm depth.

#### Multiple Drivers of Prokaryotic Diversity

**FIGURE 4:**
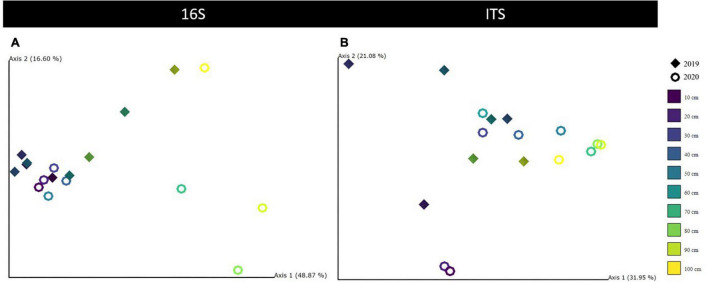
Principal coordinate analysis (PCoA) results were visualized with QIIME2 Emperor plots for 16S **(A)** and ITS **(B)** Unifrac distances along the first two axes, each depicting the variability explained in %. Diamonds are used for 2019 and rings for 2020 measurements. Different colors indicate the 10-cm depth horizons. All other soil parameters were tested and summarized in Supplementary Figure 2 (16S) and Supplementary Figure 3 (ITS).

Alpha diversity was significantly negatively correlated with soil age (ANOVA, *P* = 0.0002) and pH (ANOVA, *P* = 0.0057). Pairwise covariation revealed no significant differences between years, while diversity across all layers was significantly different (*P* < 0.05) (Table 2). As for the horizons, the deepest mineral horizon community was significantly different from all other horizons (*P* < 0.05). The beta diversity was significantly correlated with age (PERMANOVA, *P* = 0.001), horizon (PERMANOVA, *P* = 0.001), layer (PERMANOVA, *P* = 0.005), and year (PERMANOVA, *P* = 0.026). Soil moisture and organic matter content had no significant correlations (Table 2). PCoA ordination revealed that 49, 17, and 9% of all variation could be explained by the first three axes, totaling 75% (Figure 4). Samples from the top 0–60 cm horizons were visually separated from samples of the deepest horizon M2, the latter also considerably older (> 22,000 years).

**TABLE 2:** Significance tests between prokaryotic (16S) and fungal (ITS) diversity indices and soil parameters.

Parameters	Covariation	16S		ITS	
		α	β	α	β
^14^C		−0.77***	0.67***	−0.50**	0.28**
pH		−0.62**	0.05	−0.53*	0.04
Year		1.03	2.92*	0.15	2.29*
Layer		10.5**	3.66**	6.36*	2.14*
	AL–PF	6.00*		3.75	
	AL–TZ	5.36*		0.69	
	TZ–PF	4.69*		5.73*	
Horizon		11.8**	4.1***	8.51*	1.9*
	M1–M2	9.00**		3.49	
	M1–O1	2.14		2.33	
	M1–O2	0.32		1.29	
	M2–O1	4.00*		0.09	
	M2–O2	4.27*		6.00*	
	O1–O2	0.33		2	
H_2_O		0.46	0.11	0.37	0.14
SOM		0.34	0.1	0.37	0.14

Significance between alpha (α) or beta (β) diversity with soil parameters was tested with linear two-way ANOVA on 999 permutations. For pairwise covariation between layers (AL, TZ, and PF) and horizons (O1, M1, O2, M2), two-sided Kruskal–Wallis test produced the statistic H for alpha diversity values. Significance with indicated with *P < 0.05, **P < 0.01, ***P < 0.005, insignificant pairs were omitted.

#### Trends in Fungal Diversity Changes

Fungal alpha diversity was also negatively correlated with age (ANOVA, *P* = 0.0403) and pH (ANOVA, *P* = 0.0305). Significant differences for alpha diversity stemmed from the pairwise covariation between the transition zone and permafrost samples (*P* = 0.0167) and similarly between the two deepest horizons O2 and M2 (*P* = 0.0143, Table 2). Beta diversity was significantly correlated with changes of age (PERMANOVA, *P* = 0.007), layer (PERMANOVA, *P* = 0.017), horizon (PERMANOVA, *P* = 0.021), and year (PERMANOVA, *P* = 0.04). The variability of the fungal community could be explained by the first three PCoA axes with 32, 21, and 16%, respectively, although no visual separation by any environmental factor became apparent (Supplementary Figure 5).

## Discussion

Investigating microbial community responses to abrupt permafrost thawing processes is crucial to advance our understanding of ecological responses to global warming, yet it is greatly understudied (Edwards et al., 2020). There is an urgent need for linking microorganism abundances in response to increasing bioavailability with their potential to metabolize ancient permafrost carbon stocks (Turetsky et al., 2020). The findings presented enhance our understanding of cryophilic microbial biodiversity under *in situ* thermal stress within the first 2 years of an abruptly eroding thermal erosion gully in Northeast Greenland. We showed that both permafrost prokaryotes and fungi respond strongly to vertical variation within the quickly eroding permafrost habitat. Most significantly, stratification of the microbial community along organic and mineral horizons was evident, and the thaw state and the age of the soil explained the changes in diversity within the community better than other deterministic soil parameters, such as pH, organic matter content, and moisture.

### Changes in Prokaryotic Community Are Driven by Inhabited Horizons

#### Community Composition

We found Proteobacteria, Verrucomicrobiota, Bacteroidota, Actinobacteriota, Gemmatimonadota, and Acidobacteriota to be ubiquitous across all samples, in agreement with recently reviewed abundances across several Arctic sites (Malard et al., 2019). To our knowledge, only two amplicon sequencing studies have investigated the permafrost soil community in Northeast Greenland (Ganzert et al., 2014; Gittel et al., 2014b). Despite such closeness to their sampling site, we observed an overall slightly higher relative abundance of Verrucomicrobiota, Bacteroidota, and Patescibacteria as well as fewer Actinobacteriota. This might be explained by the erosion characteristics of our site, as certain abundant taxa in our study particularly occurred in the freshly thawed transition zone. Defining horizons as consisting of an organic top, underlying mineral buried topsoil, and deeper buried horizons, two studies also found a significant correlation between the horizon and microbial diversity (Frank-Fahle et al., 2014; Gittel et al., 2014b). Surprisingly, per horizon, our prokaryotic alpha diversity measurements were 1.5–2 times higher compared to their data. We hypothesized this to be simply based on varying methodological approaches, as we used ASVs instead of OTUs, increasing the number of unique sequences, which only a few permafrost erosion studies did previously (Doherty et al., 2020; Holm et al., 2020; Kirkwood et al., 2021).

#### The Permafrost Layer

At our site, several Proteobacteria classes were abundant throughout the entire profile, in agreement with previous studies (Frank-Fahle et al., 2014; Gittel et al., 2014a; Deng et al., 2015; Hultman et al., 2015; Müller et al., 2018), and explained by the metabolic diversity of those classes (Koyama et al., 2014). Abundance of Proteobacteria increased toward permafrost depths, mainly governed by the copiotrophic family Comamonadaceae formerly documented as spore formers that grew rapidly with a fresh input of labile nutrients (Fierer et al., 2007). This may explain the high relative abundance of the genera *Polaromonas*, *Sideroxydans*, *Thiobacillus*, and *Rhodoferax* in our freshly thawing permafrost samples. *Polaromonas* exclusively occurred in permafrost samples, in agreement with former studies that found this aerobic psychrophile in glacial ice and sediment environments (Darcy et al., 2011). The Gallionellaceae and Comamonadaceae families had formerly been found to dominate in several millennia-old permafrost samples (Willerslev et al., 2004; Altshuler et al., 2019; Burkert et al., 2019), confirming these taxa as part of our ancient permafrost community. In the 2019 permafrost, we found a significant abundance of *Sideroxydans*, a chemolithotroph, formerly found in peatland soils (Lipson et al., 2010; Margesin and Collins, 2019). In anaerobic subglacial lake water of Antarctica, *Polaromonas* and *Sideroxydans* were found to co-occur (Margesin, 2017).

Anaerobic degraders in permafrost were documented to include Actinobacteriota, Firmicutes, and Proteobacteria (Willerslev et al., 2004; Yergeau et al., 2010; Jansson and Taş, 2014). While in our study, Proteobacteria clearly dominated deeper mineral soils, Actinobacteriota were found mainly in 2019, but then especially in the deepest, still intact permafrost sample. Only here, also Firmicutes occurred, where they had the strongest trend as indicator species in our study. They have been found to be extremophiles persisting in intact permafrost soils (Frank-Fahle et al., 2014; Gittel et al., 2014a,b; Deng et al., 2015; Monteux et al., 2018; Tripathi et al., 2018; Burkert et al., 2019) due to their endospore-formation. We note that Firmicutes spores might be harder to extract DNA from and thereby sequence, and their higher relative abundance potentially does not reflect *in situ* intact permafrost abundance, but a response to the storage conditions.

Often, Chloroflexi taxa endemic to permafrost are not classified in databases (Jansson and Taş, 2014). We found Chloroflexi present throughout the entire soil profile, in contrast to other studies that found this taxon to dominate in permafrost samples (Frank-Fahle et al., 2014; Tuorto et al., 2014; Hultman et al., 2015). Only one other study confirmed the occurrence of Chloroflexi at surface depths (Gittel et al., 2014b). As this study was the only one within the same study area, the local biogeography could explain this trend (Gittel et al., 2014b).

#### The Thawed Soils of Transition Zone and Active Layer

Previous studies have shown the most significant changes in the microbial community to take place during long-term thawing (Elberling et al., 2013; Schostag et al., 2019). The transition from permafrost to thawed soil was among the most significant drivers of the differences in community composition in our study. This trend was typified by the increased abundance of Bacteroidota, in agreement with the literature (Frank-Fahle et al., 2014; Coolen and Orsi, 2015; Deng et al., 2015; Burkert et al., 2019), also found at the upper permafrost limit and during thawing (Müller et al., 2018; Tripathi et al., 2018). Bacteroidia were strongly represented by the Sphingobacteriales order, which include psychrotolerant non-spore-forming soil genera and lichen symbionts (Cernava et al., 2017).

In agreement with previous studies, we found Actinobacteriota (Rivkina et al., 2007; Frank-Fahle et al., 2014; Tuorto et al., 2014; Hultman et al., 2015; Mackelprang et al., 2017), Gemmatimonadota (Gittel et al., 2014a; Schostag et al., 2015; Müller et al., 2018; Altshuler et al., 2019), and Verrucomicrobiota (Gittel et al., 2014a; Deng et al., 2015; Schostag et al., 2015; Müller et al., 2018; Tripathi et al., 2018; Altshuler et al., 2019) throughout the thawed active and transition zone samples. Verrucomicrobiota are often among the first responders to seasonal thawing (Malard and Pearce, 2018) and although previously seen mainly in surface samples (Deng et al., 2015), they were the most abundant until 60 cm depth in our study. For this phylum, a relative abundance of over 20% per sample in 2020 was rarely observed previously (Schostag et al., 2015), but confirmed by the one earlier study performed in this valley (Gittel et al., 2014b). Notably, the primers used in this study have been known to indicate a slightly higher Verrucomicrobiota abundance (Takahashi et al., 2014).

Based on the utilized monitoring data, local fluctuation of surface temperature indicates almost a 60°C range. The annual freeze-up in fall supplies dead organic matter, which serves as nutrients in the next spring, when increasing temperatures and soil moisture increases after snowmelt enable microbial growth again (Jansson and Taş, 2014; Malard and Pearce, 2018). Taxa particularly adapted to this extreme and less thermally stable environment of the active layer include Acidobacteriota, Alphaproteobacteria, and Verrucomicrobiota (Deng et al., 2015; Altshuler et al., 2019). In our study, Acidobacteriota abundance decreased significantly with depth, which has been reported earlier (Frank-Fahle et al., 2014; Gittel et al., 2014b; Monteux et al., 2018; Tripathi et al., 2018; Altshuler et al., 2019), and depends on a low pH (Männistö et al., 2007; Ganzert et al., 2011). In line with this, we found that even the small changes of pH in 2019 were found significant as driver of alpha diversity in our study. Willms et al. (2021) found *Cand. Udaeobacter* as one of the most abundant soil taxa, especially in strongly acidic soils. As one of the most abundant taxa in our study, the overall acidic pH might support its high relative abundance. Malard et al. (2019) reviewed Blastocatellia abundance similar to those in our study in acidoneutral to alkaline soils, which could explain their higher abundance in our more alkaline M2 samples in 2019 as opposed to 2020. The main order in phylum in our study were Pyrinomonadales, which formerly have been observed as phototrophic lichen symbionts and are part of a rather specialist soil community. An acidoneutral soil pH, such as in most Greenland studies, was found to supply optimum microbial growth conditions (Rousk et al., 2010), which could explain the particularly high alpha diversity found across most of our samples.

#### Archaeal Abundance Within Degrading Permafrost

Archaeal abundance was marginal within the prokaryotic community composition, which is in agreement with previous findings (Yergeau et al., 2010; Rivkina et al., 2016; Mondav et al., 2017; Müller et al., 2018; Hough et al., 2020). Although we selected primers that were designed to create the least possible bias toward bacterial or archaeal taxa (Takahashi et al., 2014), they still have previously been shown to be less efficient for archaea (Parada et al., 2016). The dominance of the anaerobic, metabolically flexible Bathyarchaeia has previously been documented (Lazar et al., 2016; Xiang et al., 2017). In our study, this taxon occurred at soil depths with maximum soil moisture, supplying saturated conditions. Woesearchaeota have been documented as syntrophic partners to methanogens (Liu et al., 2018; Tveit et al., 2020), which could justify their abundance throughout the soil column, where we found several methanogenic orders, such as Methanomicrobiales, Methanosarcinales, Methanomassiliicoccales, and Methanobacteriales. Especially Methanomicrobiales have been documented in thawing permafrost (Deng et al., 2015; Wei et al., 2018). Both Euryarchaeota classes Methanomicrobiales and Methanobacteriales have been found in Greenlandic active layer soils before (Malard and Pearce, 2018). The nitrifying Nitrososphaerales oxidize ammonia, possibly explaining their abundance within the active layer where deeply rooting vegetation supplies nitrogen compounds (DeLong et al., 2014).

### Different Fungal Community in Ancient Permafrost

In our study, fungal diversity was generally lower than prokaryotic diversity and most driven by the difference of age and the thaw state of the soil. We could confirm representation of Ascomycota by Leotiomycetes and Basidiomycota by Agarimycetes in alignment with Gittel et al. (2014b), which might indicate a local pattern specific to Northeast Greenland, or even the Zackenberg valley. Ascomycota dominated our fungal community, in agreement with previous findings (Malard and Pearce, 2018; Margesin and Collins, 2019). The identified Ascomycete genera *Blumeria*, *Cystodendron*, and *Neobulgaria* of the Helotiales order are recognized as saprotrophic and plant pathogens, growing on decaying organic matter (Ekanayaka et al., 2019), as abundant in the buried organic horizon, where they occurred predominantly. Fungal abundance was commonly reported with decrease with depth in Arctic soils and is linked to a saprotrophic metabolism and the breakdown of labile carbon compounds (Gittel et al., 2014a).

### Microbial Diversity Is Driven by the Thawing of Soil Horizons

Prokaryotic alpha diversity in our study was twice as high as reported in other studies (Chu et al., 2010; Tveit et al., 2013; Frank-Fahle et al., 2014; Gittel et al., 2014b; Schostag et al., 2019), which did not detect a deeper second peak, as we did at the 40–50 cm depth in 2020. Faith’s PD values both for prokaryotic and fungal communities were comparable to one of the few permafrost studies that previously employed this index (Feng et al., 2020). A decrease of alpha diversity with depth and age has been previously shown (Tripathi et al., 2018; Burkert et al., 2019). Interestingly, utilizing relic permafrost DNA, if amplifiable, was earlier found to lead to up to 45% increased diversity estimates (Fierer, 2017). Age was a significant driver of biodiversity for both kingdoms. The clustering of samples from the youngest samples opposed to higher dispersal in samples older than 3,500 years has also been described before (Tripathi et al., 2018), and aligns with the significant age effect on diversity (Mackelprang et al., 2017; Burkert et al., 2019).

For prokaryotic diversity, each layer depicted a significantly different community, but clearly only the most significant one, the oldest horizon, was visually split in the community, as indicated by the PCoA plots. For fungi, diversity changes were driven significantly similarly driven by the age effect and the transition to the permafrost layer, indicating a strong role for the permafrost table depth of the soil rather than organic or mineral content. Furthermore, current work has suggested that physical barriers to microbial dispersal were the main driver of changes in the permafrost soil microbiomes (Bottos et al., 2018). We think the separation of the community in the study by horizons most closely depicts these barriers of habitat, hence its significance for explaining changes in community composition.

Previous studies confirmed that prokaryotic survival in permafrost is due to the occupation of viable microhabitats, such as brine channels (Gilichinsky et al., 2003) or the ability to adherence to silt or clay particles, whereas the fungal mycelium often form larger networks across rather than within the soil horizons (Jansson and Hofmockel, 2019). Jansson and Hofmockel (2019) found bacteria usually outcompeted fungi as the temperature increased, due to higher growth rates and competition for newly available nutrients. However, for all microorganisms, our results indicate the strongest community shift from frozen to thawed ground.

### Copiotrophy in Thawing Permafrost

While nutrient load and lability of organic carbon decreases within intact permafrost soils, the more stress-resilient oligotrophic (slow-growing specialist) microorganisms are usually favored over copiotrophic (fast-growing and generalist) taxa (Fierer et al., 2007). But these ecosystems are facing increased abrupt erosion until the end of this century, both globally (Turetsky et al., 2020) as well as in the Zackenberg Valley (Westermann et al., 2015; Rasmussen et al., 2018). Christensen et al. (2020) have already documented the connection between increasing events of extreme precipitation and summer temperatures, and the permafrost erosion site studied here. Habtewold et al. (2021) recently confirmed a correlation between higher carbon released from warmed Canadian soils and increased abundance of copiotrophic taxa. A former study on Alpine permafrost soil found soil warming to trigger copiotrophy (Perez-Mon et al., 2022), indicating elevated mineralization potential of increasingly bioavailable soil carbon and fresh nutrient inputs after thawing. The taxa that dominated this vulnerable zone of freshly thawed ancient carbon stocks in our study included both the copiotrophic Bacteroidia and MBNT15 phyla, as well as the oligotrophic Chthoniobacterales and Pyrinomonadales (Ho et al., 2017). But Bacteroidia were found as one of the clearest thaw zone indicator phyla and also dominated the 2020 thaw samples with more than half of all counts. They have higher carbon mineralization potential and can adapt their population size better to the available resources. In comparison, their oligotrophic counterpart Acidobacteria might be better adapted to the thermally variable active layer due to their higher stress resilience (Fierer et al., 2007). Interestingly, the abundance of copiotrophic taxa, such as the Comamonadaceae family and the often as copiotrophic classified Firmicutes even in our deepest permafrost samples (Ho et al., 2017), indicates that the short time period of storage at thawing temperatures initiated a microbial response to the abrupt thaw, both *in situ* and *in vivo*. This, clearly, indicates a need for controlled studies to verify and quantify the metabolic response of ancient permafrost taxa to thaw. Fierer et al. (2007) argued that knowledge about community composition and dominance of oligo- and copiotrophy is a crucial component for improving the prediction permafrost carbon loss due to microbial mineralization (Langille et al., 2013). Due to the quick response to readily available carbon, copiotrophic metabolism has been reviewed before to correlate with carbon release from soil ecosystems (Trivedi et al., 2013; Hurst, 2019).

### Limitations and Future Perspectives

Malard et al. (2019) and Tripathi et al. (2019) explored the description of stochastic (random and dispersal) and deterministic (biotic and abiotic selection) drivers of community composition in permafrost soils. They found top-soils to be dominated by deterministic processes, while deeper soils were controlled by stochastic processes. As permafrost soils are highly heterogenic, an assessment of these processes would help to confirm the significance of changes in this vulnerable ecosystem on its microbiome and should be considered in future efforts. Still, we can interpret from our community that higher abundance of specialist taxa in the permafrost samples indicates an importance of deterministic processes to govern, while these soils are intact.

In Arctic remote sampling sites, both core sampling techniques to reduce contamination (Bang-Andreasen et al., 2017) and temperature stable sampling and transport oppose great challenges. Due to the remote sampling site and long transport route, we decided to store the samples at 4°C and extract the DNA as fast as possible. Greater biological perturbation could have been induced with freezing and thawing. While DNA sequencing is a proxy for the total microbial community composition and not just the viable one—including taxa that might have ceased or increased abundance in response to the storage induced thaw—viability assays, incorporation, or transcriptomic studies are needed to quantify the copiotrophic taxa trends visible in our samples (Mackelprang et al., 2021). Still, taxa abundance in the deepest and most intact permafrost sample in 2019, as opposed to other depths and years, indicated the potential of depicting close to *in situ* conditions in ancient permafrost before and while thawing.

## Conclusion

While investigating the impact of abrupt permafrost thawing on the microbial community composition is usually analyzed under constraining laboratory incubations, we were the first to study an *in situ* temporal natural thaw gradient in Northeast Greenland and to show the swift adaptation potential of the ancient permafrost microbiome to abrupt erosion on a 2-year timescale and a high-resolution sampling scale, which has been rarely done previously. We found the soil age and horizons to be the most significant driver of microbial community. Furthermore, year of sampling after erosion and permafrost thaw state significantly drove microbial community composition changes with depth. Alpha diversity decreased with increasing depth, pH, and especially soil age. We were able to differentiate dominant prokaryotic taxa within the active layer, freshly thawed transition zone, and ancient permafrost samples. Finally, the transition zone, especially with increasing erosion, gave rise to copiotrophic taxa, such as Bacteroidota, indicating potential impact on the release of permafrost carbon through microbial carbon mineralization. While the universal primers we utilized were sufficient for the scope of our study, a combination with viability assays or transcriptomic studies of ancient taxa within the deepest permafrost samples could significantly improve results. These approaches are still rare across the Arctic, as recently pointed out by Mackelprang et al. (2021). They could provide a higher level of confidence about composition changes of ancient microbial taxa under thawing conditions. We recommend that future research efforts focus on the differentiation of living from dead ancient microbiomes, as well as transcriptomic approaches to elucidate how the complex process of permafrost thaw ends dormancy stages and initiates metabolic activity. These approaches could empirically elaborate the connection between microbial community changes and the globally changing climate.

## Data Availability Statement

The datasets produced and analyzed for this study can be found in the online repository: https://www.ncbi.nlm.nih.gov/bioproject/PRJNA769429, with the accession number PRJNA769429.

## Author Contributions

MS, TC, AZ, and CJ conceptualized and designed the study. MS and AZ organized the databases. MS performed the laboratory work, statistical analysis, and wrote the first draft of the manuscript. All authors contributed to manuscript revision, read, and approved the submitted version.

## Conflict of Interest

The authors declare that the research was conducted in the absence of any commercial or financial relationships that could be construed as a potential conflict of interest.

## Publisher’s Note

All claims expressed in this article are solely those of the authors and do not necessarily represent those of their affiliated organizations, or those of the publisher, the editors and the reviewers. Any product that may be evaluated in this article, or claim that may be made by its manufacturer, is not guaranteed or endorsed by the publisher.

## Footnotes

1
https://data.g-e-m.dk/

2
https://doi.org/10.17897/89G6-QD54

## References

[B1] Altshuler I. Hamel J. Turney S. Magnuson E. Lévesque R. Greer C. W. (2019). Species interactions and distinct microbial communities in high Arctic permafrost affected cryosols are associated with the CH4 and CO2 gas fluxes. *Environ. Microbiol.* 21 3711–3727. 10.1111/1462-2920.14715 31206918

[B2] AMAP (2019). *AMAP Climate Change Update 2019: an Update to Key Findings of Snow, Water, Ice and Permafrost in the Arctic (SWIPA) 2017.* Norway: Arctic Monitoring and Assessment Programme (AMAP), 12.

[B3] AMAP (2021). *Arctic Climate Change Update 2021: key Trends and Impacts. Summary for Policy-Makers.* Norway: Arctic Monitoring and Assessment Programme (AMAP). 16.

[B4] Bang-Andreasen T. Schostag M. Priemé A. Elberling B. Jacobsen C. S. (2017). Potential microbial contamination during sampling of permafrost soil assessed by tracers. . *Sci. Rep.* 7 1–11. 10.1038/srep43338 28230151 PMC5322388

[B5] Bay C. (1998). *Vegetation Mapping of the Zackenberg Valley, Northeast Greenland.* Copenhagen: Danish Polar Center & Botanical Museum, University of Copenhagen.

[B6] Bolyen E. Rideout J. R. Dillon M. R. Bokulich N. A. Abnet C. C. Al-Ghalith G. A. (2019). Reproducible, interactive, scalable and extensible microbiome data science using QIIME 2. *Nat. Biotechnol.* 37 852–857. 10.1038/s41587-019-0209-9 31341288 PMC7015180

[B7] Bottos E. M. Kennedy D. W. Romero E. B. Fansler S. J. Brown J. M. Bramer L. M. (2018). Dispersal limitation and thermodynamic constraints govern spatial structure of permafrost microbial communities. *FEMS Microbiol. Ecol.* 94 1–14. 10.1093/femsec/fiy110 29912311

[B8] Burkert A. Douglas T. A. Waldrop M. P. Mackelprang R. (2019). Changes in the Active, Dead, and Dormant Microbial Community Structure across a Pleistocene Permafrost Chronosequence. *Appl. Environ. Microbiol.* 85 1–16. 10.1128/AEM.02646-18 30683748 PMC6585489

[B9] Callahan B. J. McMurdie P. J. Holmes S. P. (2017). Exact sequence variants should replace operational taxonomic units in marker-gene data analysis. *ISME J.* 11 2639–2643. 10.1038/ismej.2017.119 28731476 PMC5702726

[B10] Cavicchioli R. Bakken L. R. Baylis M. Foreman C. M. Karl D. M. Koskella B. (2019). Scientists’ warning to humanity: microorganisms and climate change. *Nat. Rev. Microbiol.* 17 569–586. 10.1038/s41579-019-0222-5 31213707 PMC7136171

[B11] Cernava T. Erlacher A. Aschenbrenner I. A. Krug L. Lassek C. Riedel K. (2017). Deciphering functional diversification within the lichen microbiota by meta-omics. *Microbiome* 5:82. 10.1186/s40168-017-0303-5 28724401 PMC5518139

[B12] Christensen T. R. Lund M. Skov K. Abermann J. Scheller J. Scheel M. (2020). Multiple Ecosystem Effects of Extreme Weather Events in the Arctic. *Ecosystems* 24 122–136. 10.1007/s10021-020-00507-6

[B13] Christiansen H. H. (1999). Active Layer Monitoring in two Greenlandic Permafrost Areas: zackenberg and Disko Island. *Danish J. Geogr.* 99 117–121.

[B14] Christiansen H. H. Sigsgaard C. Humlum O. Rasch M. Hansen B. U. (2008). Permafrost and Periglacial Geomorphology at Zackenberg. *Adv. Ecol. Res.* 40 151–174. 10.1016/S0065-2504(07)00007-4

[B15] Chu H. Fierer N. Lauber C. L. Caporaso J. G. Knight R. Grogan P. (2010). Soil bacterial diversity in the Arctic is not fundamentally different from that found in other biomes. *Environ. Microbiol.* 12 2998–3006. 10.1111/j.1462-2920.2010.02277.x 20561020

[B16] Coolen M. J. L. Orsi W. D. (2015). The transcriptional response of microbial communities in thawing Alaskan permafrost soils. *Front. Microbiol.* 6:197. 10.3389/fmicb.2015.00197 25852660 PMC4360760

[B17] Collins T. Margesin R. (2019). Psychrophilic lifestyles: mechanisms of adaptation and biotechnological tools. *Appl. Microbiol. Biotechnol.* 103 2857–2871. 10.1007/s00253-019-09659-5 30729286

[B18] Darcy J. L. Lynch R. C. King A. J. Robeson M. S. Schmidt S. K. (2011). Global distribution of Polaromonas phylotypes – evidence for a highly successful dispersal capacity. *PLoS One* 6:e23742. 10.1371/journal.pone.0023742 21897856 PMC3163589

[B19] DeLong E. F. Lory S. Stackebrandt E. Thompson F. (eds) (2014). *The Prokaryotes – Other Major Lineages of Bacteria and the Archaea.* Germany: Springer. 10.1007/978-3-642-38954-2_329

[B20] Deng J. Yungfu G. Zhang J. Xu K. Qin Y. Yuan M. (2015). Shifts of tundra bacterial and archaeal communities along a permafrost thaw gradient in Alaska. *Mol. Ecol.* 24 222–234. 10.1111/mec.13015 25424441

[B21] Doherty S. J. Barbato R. A. Grandy A. S. Thomas W. K. Monteux S. Dorrepaal E. (2020). The Transition From Stochastic to Deterministic Bacterial Community Assembly During Permafrost Thaw Succession. *Front. Microbiol.* 11:596589. 10.3389/fmicb.2020.596589 33281795 PMC7691490

[B22] Dufrêne M. Legendre P. (1997). Species assemblages and indicator species: the need for a flexible asymmetrical approach. *Ecol. Monogr.* 67 345–366. 10.2307/2963459

[B23] Edwards A. Cameron K. A. Cook J. M. Debbonaire A. R. Furness E. Hay M. C. (2020). Microbial genomics amidst the Arctic crisis. *Microb. Genom.* 6:e000375. 10.1099/mgen.0.000375 32392124 PMC7371112

[B24] Ekanayaka A. H. Hyde K. D. Gentekaki E. McKenzie E. H. C. Zhao Q. Bulgakov T. S. (2019). Preliminary classification of Leotiomycetes. *Mycosphere* 10 310–489. 10.5943/mycosphere/10/1/7

[B25] Elberling B. Christiansen H. H. Hansen B. U. (2010). High nitrous oxide production from thawing permafrost. *Nat. Geosci.* 3 332–335. 10.1038/ngeo803

[B26] Elberling B. Michelsen A. Schädel C. Schuur E. A. G. Christiansen H. H. Berg L. (2013). Long-term CO2 production following permafrost thaw. *Nat. Clim. Chang.* 3 890–894. 10.1038/nclimate1955

[B27] Elberling B. Tamstorf M. P. Michelsen A. Arndal M. F. Sigsgaard C. Illeris L. (2008). Soil and Plant Community-Characteristics and Dynamics at Zackenberg. *Adv. Ecol. Res.* 40 223–248. 10.1016/S0065-2504(07)00010-4

[B28] Feng J. Wang C. Lei J. Yang Y. Yan Q. Zhou X. (2020). Warming-induced permafrost thaw exacerbates tundra soil carbon decomposition mediated by microbial community. *Microbiome* 8:3. 10.1186/s40168-019-0778-3 31952472 PMC6969446

[B29] Fierer N. (2017). Embracing the unknown: disentangling the complexities of the soil microbiome. *Nat. Rev. Microbiol.* 15 579–590. 10.1038/nrmicro.2017.87 28824177

[B30] Fierer N. Bradford M. A. Jackson R. B. (2007). Toward an ecological classification of soil bacteria. *Ecology* 88 1354–1364. 10.1007/s00209-018-2115-0 17601128

[B31] Frank-Fahle B. A. Yergeau É Greer C. W. Lantuit H. Wagner D. (2014). Microbial functional potential and community composition in permafrost-affected soils of the NW Canadian Arctic. *PLoS One* 9:e84761. 10.1371/journal.pone.0084761 24416279 PMC3885591

[B32] Ganzert L. Bajerski F. Wagner D. (2014). Bacterial community composition and diversity of five different permafrost-affected soils of Northeast Greenland. *FEMS Microbiol. Ecol.* 89 426–441. 10.1111/1574-6941.12352 24819653

[B33] Ganzert L. Lipski A. Hubberten H.-W. Wagner D. (2011). The impact of different soil parameters on the community structure of dominant bacteria from nine different soils located on Livingston Island, South Shetland Archipelago, Antarctica. *FEMS Microbiol. Ecol.* 76 476–491. 10.1111/j.1574-6941.2011.01068.x 21314705

[B34] Gaylarde C. Ogawa A. Beech I. Kowalski M. Baptista-Neto J. A. (2017). Analysis of dark crusts on the church of Nossa Senhora do Carmo in Rio de Janeiro, Brazil, using chemical, microscope and metabarcoding microbial identification techniques. *Int. Biodeterior. Biodegrad.* 117 60–67. 10.1016/j.ibiod.2016.11.028

[B35] Gilichinsky D. Rivkina E. Shcherbakova V. Laurinavichuis K. Tiedje J. (2003). Supercooled water brines within permafrost – An unknown ecological niche for microorganisms: a model for astrobiology. *Astrobiology* 3 331–341. 10.1089/153110703769016424 14577882

[B36] Gittel A. Bárta J. Kohoutová I. Mikutta R. Owens S. Gilbert J. (2014a). Distinct microbial communities associated with buried soils in the Siberian tundra. *ISME J.* 8 841–853. 10.1038/ismej.2013.219 24335828 PMC3960545

[B37] Gittel A. Bárta J. Kohoutová I. Schnecker J. Wild B. Čapek P. (2014b). Site- and horizon-specific patterns of microbial community structure and enzyme activities in permafrost-affected soils of Greenland. *Front. Microbiol.* 5:541. 10.3389/fmicb.2014.00541 25360132 PMC4199454

[B38] Habtewold J. Z. Helgason B. L. Yanni S. F. Janzen H. H. Ellert B. H. Gregorich E. G. (2021). Warming effects on the structure of bacterial and fungal communities in diverse soils. *Appl. Soil Ecol.* 163:103973. 10.1016/j.apsoil.2021.103973

[B39] Hansen B. U. L. F. Sigsgaard C. Rasmussen L. Cappelen J. Hinkler J. Mernild S. H. (2008). “Present – Day Climate at Zackenberg. *Adv. Ecol. Res.* 40 111–49. 10.1016/S0065-2504(07)00006-2

[B40] Hayes D. J. Kicklighter D. W. McGuire A. D. Chen M. Zhuang Q. Yuan F. (2014). The impacts of recent permafrost thaw on land-atmosphere greenhouse gas exchange. *Environ. Res. Lett.* 9:045005. 10.1088/1748-9326/9/4/045005

[B41] Ho A. Di Lonardo D. P. Bodelier P. L. E. (2017). Revisiting life strategy concepts in environmental microbial ecology. *FEMS Microbiol. Ecol.* 93 1–14. 10.1093/femsec/fix006 28115400

[B42] Hollesen J. Elberling B. Jansson P. E. (2011). Future active layer dynamics and carbon dioxide production from thawing permafrost layers in Northeast Greenland. *Glob. Chang. Biol.* 17 911–926. 10.1111/j.1365-2486.2010.02256.x

[B43] Holm S. Walz J. Horn F. Yang S. Grigoriev M. N. Wagner D. (2020). Methanogenic response to long-term permafrost thaw is determined by paleoenvironment. *FEMS Microbiol. Ecol.* 96 1–13. 10.1093/femsec/fiaa021 32031215 PMC7046019

[B44] Hough M. McClure A. Bolduc B. Dorrepaal E. Saleska S. Klepac-Ceraj V. (2020). Biotic and Environmental Drivers of Plant Microbiomes Across a Permafrost Thaw Gradient. *Front. Microbiol.* 11:796. 10.3389/fmicb.2020.00796 32499761 PMC7243355

[B45] Hultman J. Waldrop M. P. Mackelprang R. David M. M. McFarland J. Blazewicz S. J. (2015). Multi-omics of permafrost, active layer and thermokarst bog soil microbiomes. *Nature* 521 208–212. 10.1038/nature14238 25739499

[B46] Hurst C. J. (ed.) (2019). *Understanding Terrestrial Microbial Communities.* Berlin: Springer. 10.1007/978-3-10777-2

[B47] IPCC (2019). “Global Warming of 1.5^°^C. An IPCC Special Report on the impacts of global warming of 1.5^°^C above pre-industrial levels and related global greenhouse gas emission pathways” in *The Context of Strengthening the Global Response to the Threat of Climate Change, Sustainable Development, and Efforts to Eradicate Poverty.* eds Masson-Delmotte V. Zhai P. Pörtner H.-O. Roberts D. Skea J. Shukla P. R. (Switzerland: IPCC).

[B48] IPCC (2021). “Summary for Policymakers” in *Climate Change 2021: the Physical Science Basis. Contribution of Working Group I to the Sixth Assessment Report of the Intergovernmental Panel on Climate Change.* Edn. eds Masson-Delmotte V. Zhai P. Pirani A. Connors S. L. Péan C. Berger S. (Cambridge: Cambridge University Press).

[B49] Jansson J. K. Hofmockel K. S. (2019). Soil microbiomes and climate change. *Nat. Rev. Microbiol.* 18 35–46. 10.1038/s41579-019-0265-7 31586158

[B50] Jansson J. K. Taş N. (2014). The microbial ecology of permafrost. *Nat. Rev. Microbiol.* 12 414–425. 10.1038/nrmicro3262 24814065

[B51] Johnson S. S. Hebsgaard M. B. Christensen T. R. Mastepanov M. Nielsen R. Munch K. (2007). Ancient bacteria show evidence of DNA repair. *PNAS* 105 10631–10631. 10.1073/pnas.0710637105 PMC1958816 17728401

[B52] Kirkwood J. A. H. Roy-Léveillée P. Mykytczuk N. Packalen M. McLaughlin J. Laframboise A. (2021). Soil Microbial Community Response to Permafrost Degradation in Palsa Fields of the Hudson Bay Lowlands: implications for Greenhouse Gas Production in a Warming Climate. *Global Biogeochem. Cycles* 35:e2021GB006954. 10.1029/2021GB006954

[B53] Koyama A. Wallenstein M. D. Simpson R. T. Moore J. C. (2014). Soil bacterial community composition altered by increased nutrient availability in Arctic tundra soils. *Front. Microbiol.* 5:516. 10.3389/fmicb.2014.00516 25324836 PMC4183186

[B54] Langille M. G. I. Zaneveld J. Caporaso J. G. McDonald D. Knights D. Reyes J. A. (2013). Predictive functional profiling of microbial communities using 16S rRNA marker gene sequences. *Nat. Biotechnol.* 31 814–821. 10.1038/nbt.2676 23975157 PMC3819121

[B55] Lazar C. S. Baker B. J. Seitz K. Hyde A. S. Dick G. J. Hinrichs K. U. (2016). Genomic evidence for distinct carbon substrate preferences and ecological niches of *Bathyarchaeota* in estuarine sediments. *Environ. Microbiol.* 18 1200–1211. 10.1111/1462-2920.13142 26626228

[B56] Levin I. Kromer B. (2004). The tropospheric 14CO2 level in mid-latitudes of the Northern Hemisphere (1959-2003). *Radiocarbon* 46 1261–1272. 10.2458/azu_js_rc.46.4181 30854509

[B57] Lim P. P. Pearce D. A. Convey P. Lee L. S. Chan K. G. Tan G. Y. A. (2020). Effects of freeze-thaw cycles on High Arctic soil bacterial communities. *Polar Sci.* 23:100487. 10.1016/j.polar.2019.100487

[B58] Lipson D. A. Jha M. Raab T. K. Oechel W. C. (2010). Reduction of iron (III) and humic substances plays a major role in anaerobic respiration in an Arctic peat soil. *J. Geophys. Res.* 115 1–13. 10.1029/2009JG001147

[B59] Liu X. Li M. Castelle C. J. Probst A. J. Zhou A. Pan J. (2018). Insights into the ecology, evolution, and metabolism of the widespread Woesearchaeotal lineages. *Microbiome* 6:102. 10.1186/s40168-018-0488-2 29884244 PMC5994134

[B60] Mackelprang R. Burkert A. Haw M. Mahendrarajah T. Conaway C. H. Douglas T. A. (2017). Microbial survival strategies in ancient permafrost: insights from metagenomics. *ISME J.* 11 2305–2318. 10.1038/ismej.2017.93 28696425 PMC5607373

[B61] Mackelprang R. Saleska S. R. Jacobsen C. S. Jansson J. K. Taş N. (2016). Permafrost Meta-Omics and Climate Change. *Annu. Rev. Earth Planet. Sci.* 44 439–462. 10.1146/annurev-earth-060614-105126

[B62] Mackelprang R. Tas N. Waldrop M. (2021). “2 Functional response of microbial communities to permafrost thaw” in *Microbial Life in the Cryosphere and Its Feedback on Global Change.* eds Liebner S. Ganzert L. (Berlin: De Gruyter). 27–42. 10.1515/9783110497083-002

[B63] Malard L. A. Pearce D. A. (2018). Microbial diversity and biogeography in Arctic soils. *Environ. Microbiol. Rep.* 10 611–625. 10.1111/1758-2229.12680 30028082

[B64] Malard L. A. Anwar M. Z. Jacobsen C. S. Pearce A. Pearce D. A. Pearce A. (2019). Biogeographical patterns in soil bacterial communities across the Arctic region. *FEMS Microbiol. Ecol.* 95:fiz128. 10.1101/655431 PMC6736398 31429869

[B65] Männistö M. K. Tiirola M. Häggblom M. M. (2007). Bacterial communities in Arctic fjelds of Finnish Lapland are stable but highly pH-dependent. *FEMS Microbiol. Ecol.* 59 452–465. 10.1111/j.1574-6941.2006.00232.x 17328122

[B66] Margesin R. (2009). “Permafrost Soils” in *Soil Biology.* 4th Edn. ed. Margesin R. (Berlin: Springer-Verlag). 10.1007/978-3-540-69371-0

[B67] Margesin R. (2017). *Psychrophiles: from Biodiversity to Biotechnology.* 2nd edition. Berlin: Springer-Verlag. 10.1007/978-3-319-57057-0.

[B68] Margesin R. Collins T. (2019). Microbial ecology of the cryosphere (glacial and permafrost habitats): current knowledge. *Appl. Microbiol. Biotechnol.* 103 2537–2549. 10.1007/s00253-019-09631-3 30719551 PMC6443599

[B69] Martin M. (2011). Cutadapt removes adapter sequences from high-throughput sequencing reads. *EMBnet.J.* 17:10. 10.14806/ej.17.1.200

[B70] Metcalfe D. B. Hermans T. D. G. Ahlstrand J. Becker M. Berggren M. Björk R. G. (2018). Patchy field sampling biases understanding of climate change impacts across the Arctic. *Nat. Ecol. Evol.* 2 1443–1448. 10.1038/s41559-018-0612-5 30013133

[B71] Mondav R. McCalley C. K. Hodgkins S. B. Frolking S. Saleska S. R. Rich V. I. (2017). Microbial network, phylogenetic diversity and community membership in the active layer across a permafrost thaw gradient. *Environ. Microbiol.* 19 3201–3218. 10.1111/1462-2920.13809 28574203

[B72] Monteux S. Weedon J. T. Gavazov G. B. K. Blume-Werry G. Gavazov K. Jassey V. E. J. (2018). Long-term in situ permafrost thaw effects on bacterial communities and potential aerobic respiration. *ISME J.* 12 2129–2141. 10.1038/s41396-018-0176-z 29875436 PMC6092332

[B73] Müller O. Bang-Andreasen T. White R. A. Elberling B. Taş N. Kneafsey T. (2018). Disentangling the complexity of permafrost soil by using high resolution profiling of microbial community composition, key functions and respiration rates. *Environ. Microbiol.* 20 4328–4342. 10.1111/1462-2920.14348 29971895

[B74] Nilsson R. H. Larsson K. Taylor A. F. S. Bengtsson-Palme J. Jeppesen T. S. Schigel D. (2019). The UNITE database for molecular identification of fungi: handling dark taxa and parallel taxonomic classifications. *Nucleic Acids Res.* 47 D259–D264. 10.1093/nar/gky1022 30371820 PMC6324048

[B75] Oksanen J. Blanchet G. Friendly M. Kindt R. Legendre P. McGlinn D. (2019). *vegan: Community Ecology Package.* Available online at: https://CRAN.R-project.org/package=vegan

[B76] Panikov N. S. Flanagan P. W. Oechel W. C. Mastepanov M. A. Christensen T. R. (2006). Microbial activity in soils frozen to below −39^°^C. *Soil Biol. Biochem.* 38 785–794. 10.1016/j.soilbio.2005.07.004

[B77] Parada A. E. Needham D. M. Fuhrman J. A. (2016). Every base matters: assessing small subunit rRNA primers for marine microbiomes with mock communities, time series and global field samples. *Environ. Microbiol.* 18 1403–1414. 10.1111/1462-2920.13023 26271760

[B78] Pedregosa F. Varoquaux G. Gramfort A. Michel V. Thirion B. Grisel O. (2011). Scikit-learn: machine Learning in Python. *J. Mach. Learn. Res.* 12 2825–2830. 10.1080/13696998.2019.1666854 31505982

[B79] Perez-Mon C. Stierli B. Plötze M. Frey B. (2022). Fast and persistent responses of alpine permafrost microbial communities to in situ warming. *Sci. Total Environ.* 807:150720. 10.1016/j.scitotenv.2021.150720 34610405

[B80] Price M. N. Dehal P. S. Arkin A. P. (2010). FastTree 2 – Approximately Maximum-Likelihood Trees for Large Alignments. *PLoS One* 5:e9490. 10.1371/journal.pone.0009490 20224823 PMC2835736

[B81] Quast C. Pruesse E. Yilmaz P. Gerken J. Schweer T. Glo F. O. (2013). The SILVA ribosomal RNA gene database project: improved data processing and web-based tools. *Nucleic Acids Res.* 41 590–596. 10.1093/nar/gks1219 23193283 PMC3531112

[B82] R Core Team (2021). *R: A Language and Environment for Statistical Computing.* Vienna: R Foundation for Statistical Computing.

[B83] R Studio Team (2021). *RStudio: Integrated Development Environment for R.* Boston, MA: RStudio, Inc.

[B84] Rasmussen L. H. Zhang W. Hollesen J. Cable S. Christiansen H. H. Jansson P. E. (2018). Modelling present and future permafrost thermal regimes in Northeast Greenland. *Cold Reg. Sci. Technol.* 146 199–213. 10.1016/j.coldregions.2017.10.011

[B85] Reimer P. J. Edouard Bard B. Alex Bayliss B. Warren Beck B. J. Paul Blackwell B. G. Christopher Bronk (2013). Intcal13 and Marine13 Radiocarbon Age Calibration Curves 0–50,000 Years Cal Bp. *Radiocarbon* 55 1869–1887. 10.2458/azu_js_rc.55.16947 30854509

[B86] Rivkina E. Petrovskaya L. Vishnivetskaya T. Krivushin K. Shmakova L. Tutukina M. (2016). Metagenomic analyses of the late Pleistocene permafrost – Additional tools for reconstruction of environmental conditions. *Biogeosciences* 13 2207–2219. 10.5194/bg-13-2207-2016

[B87] Rivkina E. Shcherbakova V. Laurinavichius K. Petrovskaya L. Krivushin K. Kraev G. (2007). Biogeochemistry of methane and methanogenic archaea in permafrost. *FEMS Microbiol. Ecol.* 61 1–15. 10.1111/j.1574-6941.2007.00315.x 17428301

[B88] Rousk J. Bååth E. Brookes P. C. Lauber C. L. Lozupone C. Caporaso J. G. (2010). Soil bacterial and fungal communities across a pH gradient in an arable soil. *ISME J.* 4 1340–1351. 10.1038/ismej.2010.58 20445636

[B89] Schostag M. Priemé A. Jacquiod S. Russel J. Ekelund F. Jacobsen C. S. (2019). Bacterial and protozoan dynamics upon thawing and freezing of an active layer permafrost soil. *ISME J.* 13 1345–1359. 10.1038/s41396-019-0351-x 30692629 PMC6474304

[B90] Schostag M. Stibal M. Jansson J. K. Jacobsen C. S. Stibal M. Priemé A. (2015). Distinct summer and winter bacterial communities in the active layer of Svalbard permafrost revealed by DNA- and RNA-based analyses. *Front. Microbiol.* 6:399. 10.3389/fmicb.2015.00399 25983731 PMC4415418

[B91] Schuur E. A. G. Mack M. C. (2018). Ecological Response to Permafrost Thaw and Consequences for Local and Global Ecosystem Services. *Annu. Rev. Ecol. Evol. Syst.* 49 279–301. 10.1146/annurev-ecolsys-121415-032349

[B92] Schuur E. A. G. McGuire A. D. Schädel C. Grosse G. Harden J. W. Hayes D. J. (2015). Climate change and the permafrost carbon feedback. *Nature* 520 171–179. 10.1038/nature14338 25855454

[B93] Takahashi S. Tomita J. Nishioka K. Hisada T. Nishijima M. (2014). Development of a prokaryotic universal primer for simultaneous analysis of Bacteria and Archaea using next-generation sequencing. *PLoS One* 9:e105592. 10.1371/journal.pone.0105592 25144201 PMC4140814

[B94] Tarnocai C. Canadell J. G. Schuur E. A. G. G. Kuhry P. Mazhitova G. Zimov S. (2009). Soil organic carbon pools in the northern circumpolar permafrost region. *Global Biogeochem. Cycles* 23 1–11. 10.1029/2008GB003327 30683748

[B95] Tripathi B. M. Kim H. M. Jung J. Y. Nam S. Hyeon T. J. Kim M. (2019). Distinct taxonomic and functional profiles of the microbiome associated with different soil horizons of a moist tussock tundra in Alaska. *Front. Microbiol.* 10:1442. 10.3389/fmicb.2019.01442 31316487 PMC6610311

[B96] Tripathi B. M. Kim M. Kim Y. Byun E. Yang J. W. Ahn J. (2018). Variations in bacterial and archaeal communities along depth profiles of Alaskan soil cores. *Nat. Sci. Reports* 8:504. 10.1038/s41598-017-18777-x 29323168 PMC5765012

[B97] Trivedi P. Anderson I. C. Singh B. K. (2013). Microbial modulators of soil carbon storage: integrating genomic and metabolic knowledge for global prediction. *Trends Microbiol.* 21 641–651. 10.1016/j.tim.2013.09.005 24139848

[B98] Tuorto S. J. Darias P. McGuinness L. R. Panikov N. Zhang T. Häggblom M. M. (2014). Bacterial genome replication at subzero temperatures in permafrost. *ISME J.* 8 139–149. 10.1038/ismej.2013.140 23985750 PMC3869017

[B99] Turetsky M. Abbott B. Jones M. Walter Anthony K. M. Olefeldt D. Schuur E. A. G. (2020). Carbon release through abrupt permafrost thaw. *Nat. Geosci.* 13 138–143. 10.1038/s41561-019-0526-0

[B100] Tveit A. T. Kiss A. Winkel M. Horn F. Hájek T. Svenning M. M. (2020). Environmental patterns of brown moss- and Sphagnum-associated microbial communities. *Sci Rep.* 10:22412. 10.1038/s41598-020-79773-2 33376244 PMC7772339

[B101] Tveit A. T. Schwacke R. Svenning M. M. Urich T. (2013). Organic carbon transformations in high-Arctic peat soils: key functions and microorganisms. *ISME J.* 7 299–311. 10.1038/ismej.2012.99 22955232 PMC3554415

[B102] Wei S. Cui H. Zhu Y. Lu Z. Pang S. Zhang S. (2018). Shifts of methanogenic communities in response to permafrost thaw results in rising methane emissions and soil property changes. *Extremophiles* 22 447–459. 10.1007/s00792-018-1007-x 29429010

[B103] Westermann S. Elberling B. Højlund Pedersen S. Stendel M. Hansen B. U. Liston G. E. (2015). Future permafrost conditions along environmental gradients in Zackenberg. *Greenland. Cryosphere* 9 719–735. 10.5194/tc-9-719-2015

[B104] Willerslev E. Hansen A. J. Rønn R. Brand T. B. Barnes I. Wiuf C. (2004). Long-term persistence of bacterial DNA. *Curr. Biol.* 14 13–14. 10.1016/j.cub.2003.12.012 14711425

[B105] Willms I. M. Bolz S. H. Yuan J. Krafft L. Schneider D. Schöning I. (2021). The ubiquitous soil verrucomicrobial clade ‘Candidatus Udaeobacter’ shows preferences for acidic pH. *Environ. Microbiol. Rep.* 13 878–883. 10.1111/1758-2229.13006 34459151

[B106] Xiang X. Wang R. Wang H. Gong L. Man B. Xu Y. (2017). Distribution of *Bathyarchaeota* communities across different terrestrial settings and their potential ecological functions. *Sci. Rep.* 7:45028. 10.1038/srep45028 28322330 PMC5359579

[B107] Yergeau E. Hogues H. Whyte L. G. Greer C. W. (2010). The functional potential of high Arctic permafrost revealed by metagenomic sequencing, qPCR and microarray analyses. *ISME J.* 4 1206–1214. 10.1038/ismej.2010.41 20393573

