# Supplementary material for: Microbial Community Changes in 26,500-Year-Old Thawing Permafrost
Source: Front Microbiol. 2022 Mar 24;13:787146. doi: 10.3389/fmicb.2022.787146 (PMC8988141; doi:10.3389/fmicb.2022.787146)
Supplement: Supplementary file 1 [file Table_1.DOCX]

## Supplementary material


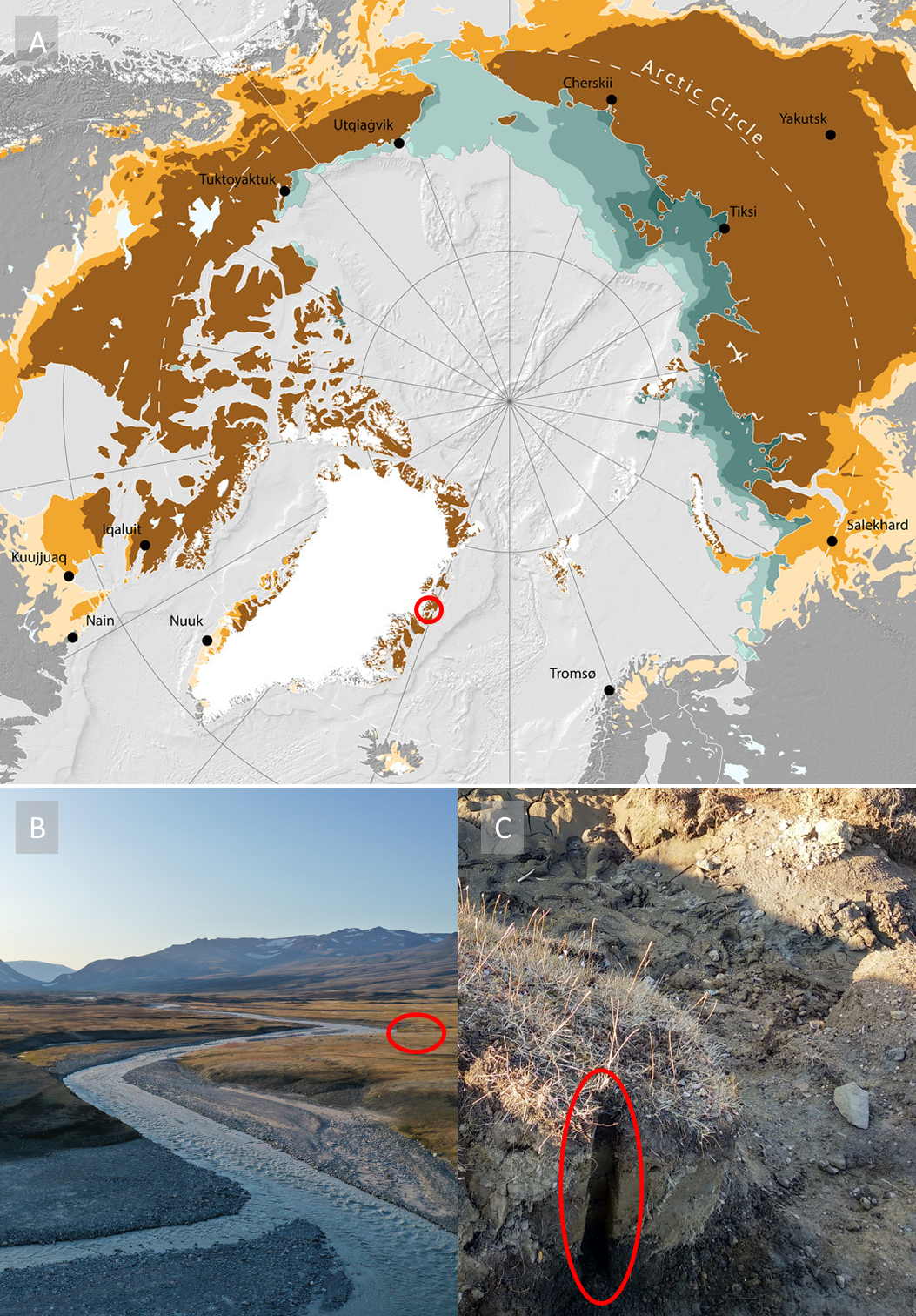


Supplementary Figure 1. (**A**) Sampling map including map of the Arctic Circle (modified from “Permafrost in the Northern Hemisphere” 2020 by GRIDA) with Zackenberg, NE Greenland, circled in red. (**B**) View on Zackenberg Valley and river, thermal permafrost erosion site circled in red. (**C**) Within the erosion site, samples of this study were taken as indicated in red.


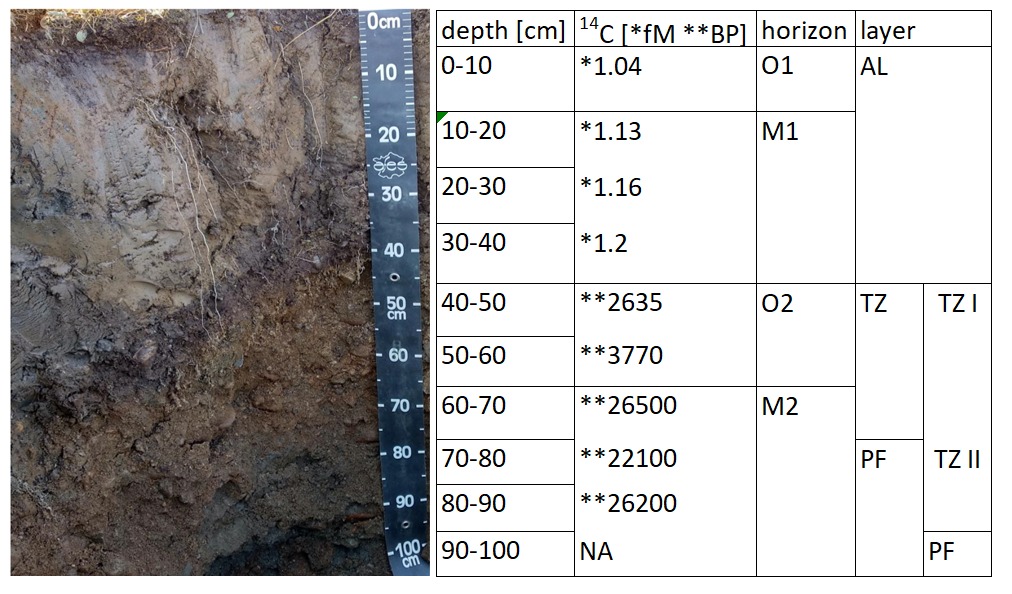


Supplementary Figure 2. Left: Photo of the sampling site and visible soil horizons. Right: Table including the radiocarbon dates in fM and BP. Based on these ages, the horizons O1, M1, O2, M2 were formulated in agreement with SOM contents of soils (O = organic, M = mineral). The layers AL, TZ and PF stand for active layer, transition (thaw) zone and permafrost.


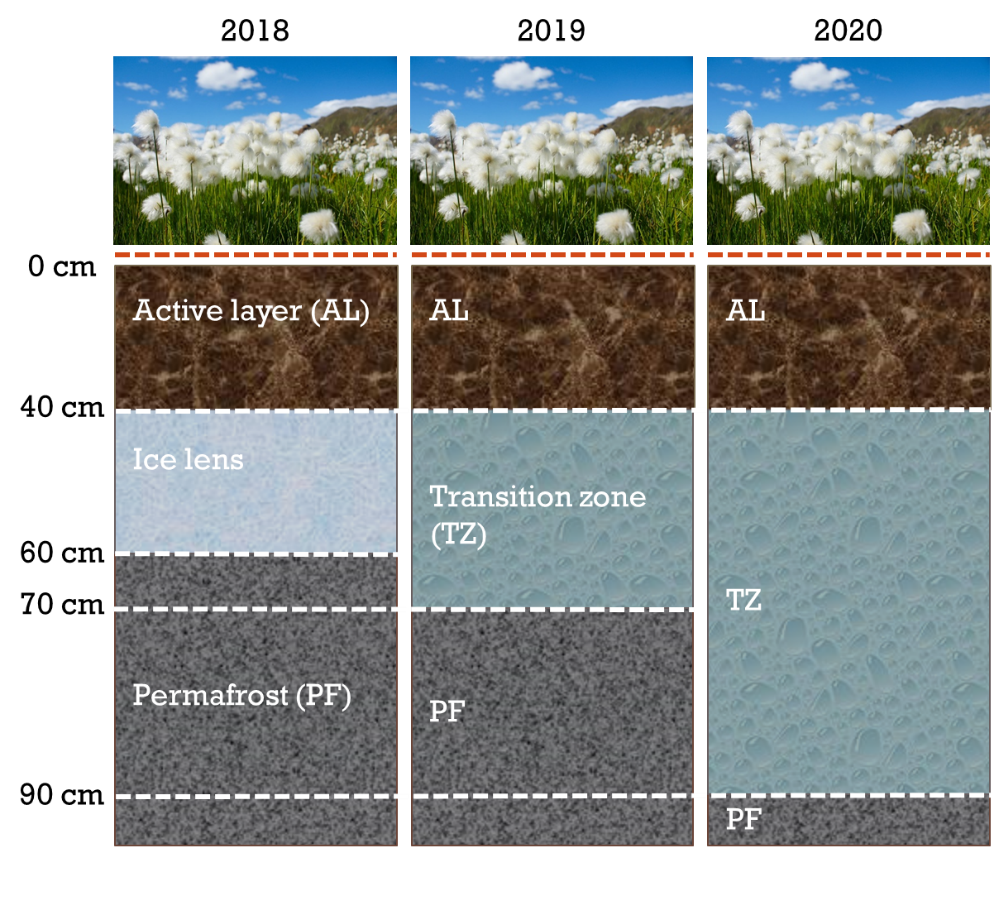


Supplementary Figure 3. Scheme of local active layer deepening at abrupt permafrost erosion site in Zackenberg, NE Greenland. The collapse of a formerly existing thermokarst took place in 2018, revealing a 20 cm thick ice lens. The active layer (AL) thickness increased from 40 cm in 2018 to 70 cm deep material in 2019 after the ice lens melted, down to 90 cm depth in 2020. The newly thawed material is labelled transition zone (TZ), while the ground remaining frozen is still frozen permafrost (PF).

Amplification protocol:

With a first PCR, utilizing 2x PCRBIO Ultra Mix, samples were initialized at 95 °C for 2 min., following 33 cycles of denaturation at 95 °C 15s, annealing at 55 °C 15s and elongation at 68 °C 40s for exponential amplification and get finalized cooling down to 68 °C for 4 min. The second PCR was run on the PCR I products, multiplexing primers and 2x PCRBIO Ultra Mix with the program: initializing at 95 °C for 1 min., following 13 cycles of denaturation at 98 °C 10s, annealing at 55 °C 20s, elongation at 68 °C 40s for exponential amplification and cooling down to 68 °C for 5 min.

Supplementary Table 1. Sequencing, filtering statistics, and alpha diversity indices for 16S and ITS sequences. Raw reads included all reads supplied by Illumina MiSeq sequencing, filtered reads were non-chimeric reads that passed filtering, denoising, merging of raw reads in %. Alpha diversity included amplicon sequence variants (ASVs), Faith’s Phylogenetic Diversity (Faith PD), Shannon index, ACE index, Pielou’s Evenness (Pielou), and Fisher index. For instances with “NA”, no sufficient reads were left for downstream analyses and samples were omitted.

| year | depth | 16S | | | | | | | | ITS | | | | | | | |
| --- | --- | --- | --- | --- | --- | --- | --- | --- | --- | --- | --- | --- | --- | --- | --- | --- | --- |
|  |  | Raw reads | Filtered reads (%) | ASVs | Faith PD | Shannon | ACE | Pielou | Fisher | Raw reads | Filtered reads (%) | ASVs | Faith PD | Shannon | ACE | Pielou | Fisher |
| 2019 | 0-10 | 763437 | 47.72 | 4897 | 259 | 11.1 | 4906 | 0.9 | 800 | NA | NA | NA | NA | NA | NA | NA | NA |
|  | 10-20 | 593053 | 51.26 | 2897 | 200 | 8.8 | 2903 | 0.77 | 444 | 53916 | 26.19 | 107 | 39 | 4.2 | 107 | 0.63 | 16 |
|  | 20-30 | 955718 | 53.47 | 2509 | 186 | 8 | 2516 | 0.7 | 343 | 106815 | 53.75 | 95 | 37 | 2.6 | 95 | 0.4 | 11 |
|  | 30-40 | 610675 | 52.96 | 2352 | 177 | 8.4 | 2357 | 0.75 | 343 | 98774 | 59.57 | 301 | 88 | 4.9 | 301 | 0.59 | 41 |
|  | 40-50 | 586017 | 54.81 | 2019 | 164 | 7.9 | 2024 | 0.72 | 288 | 279657 | 38.73 | 318 | 82 | 5.4 | 318 | 0.65 | 40 |
|  | 50-60 | 833210 | 46.18 | 1762 | 153 | 7.7 | 1770 | 0.71 | 239 | 163966 | 59.65 | 239 | 73 | 5.3 | 239 | 0.67 | 29 |
|  | 60-70 | 657450 | 53.66 | 1820 | 158 | 7.5 | 1823 | 0.69 | 251 | NA | NA | NA | NA | NA | NA | NA | NA |
|  | 70-80 | 992317 | 60.5 | 836 | 70 | 7.6 | 837 | 0.79 | 96 | 24340 | 25.88 | 24 | 10 | 3.9 | 24 | 0.85 | 3 |
|  | 80-90 | 561828 | 63.53 | 418 | 33 | 5.8 | 418 | 0.66 | 47 | 29224 | 53.11 | 27 | 7 | 3.4 | 27 | 0.7 | 3 |
| 2020 | 0-10 | 379162 | 9.92 | 3371 | 314 | 10.2 | 3477 | 0.87 | 897 | 116977 | 36.87 | 114 | 34 | 3.6 | 114 | 0.53 | 14 |
|  | 10-20 | 442462 | 11.49 | 2984 | 281 | 9.2 | 3051 | 0.47 | 692 | 101791 | 57.81 | 156 | 49 | 1.6 | 156 | 0.22 | 19 |
|  | 20-30 | 297831 | 12.57 | 2040 | 216 | 8.5 | 2104 | 0.79 | 463 | 49939 | 50.08 | 237 | 71 | 5.2 | 237 | 0.66 | 36 |
|  | 30-40 | 419611 | 12.15 | 2988 | 289 | 9.3 | 3060 | 0.77 | 693 | 38743 | 50.29 | 287 | 81 | 6.6 | 287 | 0.81 | 48 |
|  | 40-50 | 514732 | 14.99 | 3740 | 339 | 9.3 | 3842 | 0.8 | 821 | 26392 | 48.32 | 233 | 74 | 5.8 | 233 | 0.73 | 40 |
|  | 50-60 | NA | NA | NA | NA | NA | NA | NA | NA | 85392 | 58.01 | 228 | 64 | 3.8 | 228 | 0.48 | 31 |
|  | 60-70 | 404303 | 14.46 | 1625 | 161 | 7.4 | 1655 | 0.78 | 310 | 87414 | 63.87 | 224 | 61 | 3.4 | 224 | 0.43 | 30 |
|  | 70-80 | 490505 | 19.17 | 1473 | 137 | 6.2 | 1490 | 0.7 | 248 | 97010 | 63.22 | 237 | 58 | 4.4 | 237 | 0.56 | 31 |
|  | 80-90 | 385277 | 20.87 | 722 | 66 | 4.5 | 730 | 0.59 | 109 | 94842 | 63.98 | 183 | 45 | 4.1 | 183 | 0.55 | 23 |
|  | 90-100 | 369322 | 18.09 | 502 | 48 | 4.2 | 506 | 0.48 | 74 | 88064 | 64.02 | 178 | 38 | 5.2 | 178 | 0.69 | 23 |


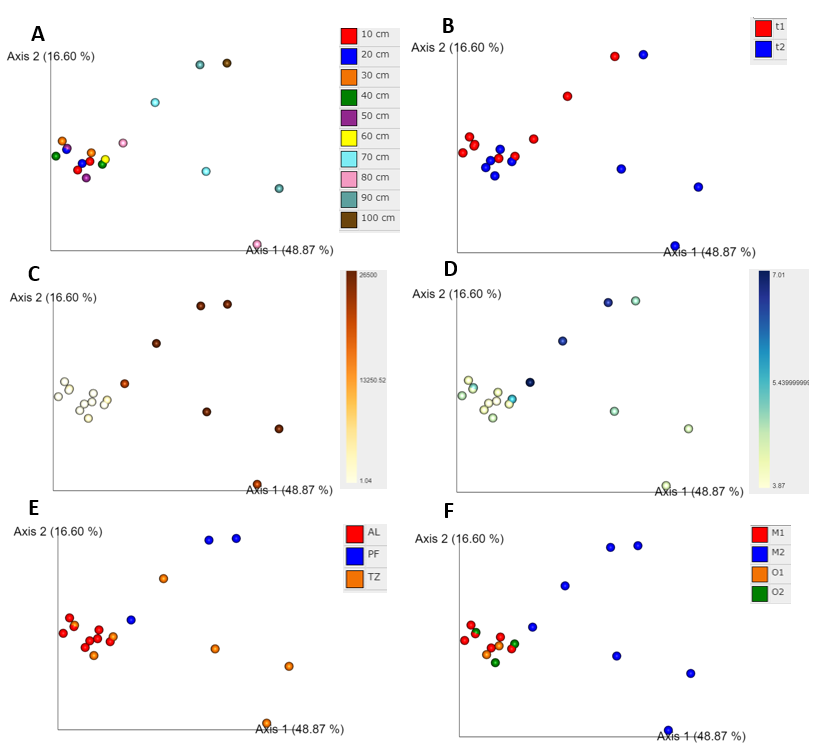


Supplementary Figure 4. Principal coordinate analysis (PCoA) results were visualized with QIIME2 Emperor plots for 16S Unifrac distances along the first two axes, each depicting the variability explained in %. Diamonds are used for 2019 and rings for 2020 measures. The graphs depict various soil parameter and their legend on the left, including depth (A), year (B), soil age ^14^C (C), pH (D), layer (E) and horizon (F). Abbreviations used herein include t1 for 2019, t2 for 2020, AL for active layer, PF for permafrost, TZ for transition zone, O1 for surface organic layer, M1 for top mineral layer, O2 for buried organic layer and M2 for ancient mineral layer, as defined in Tab. 1.


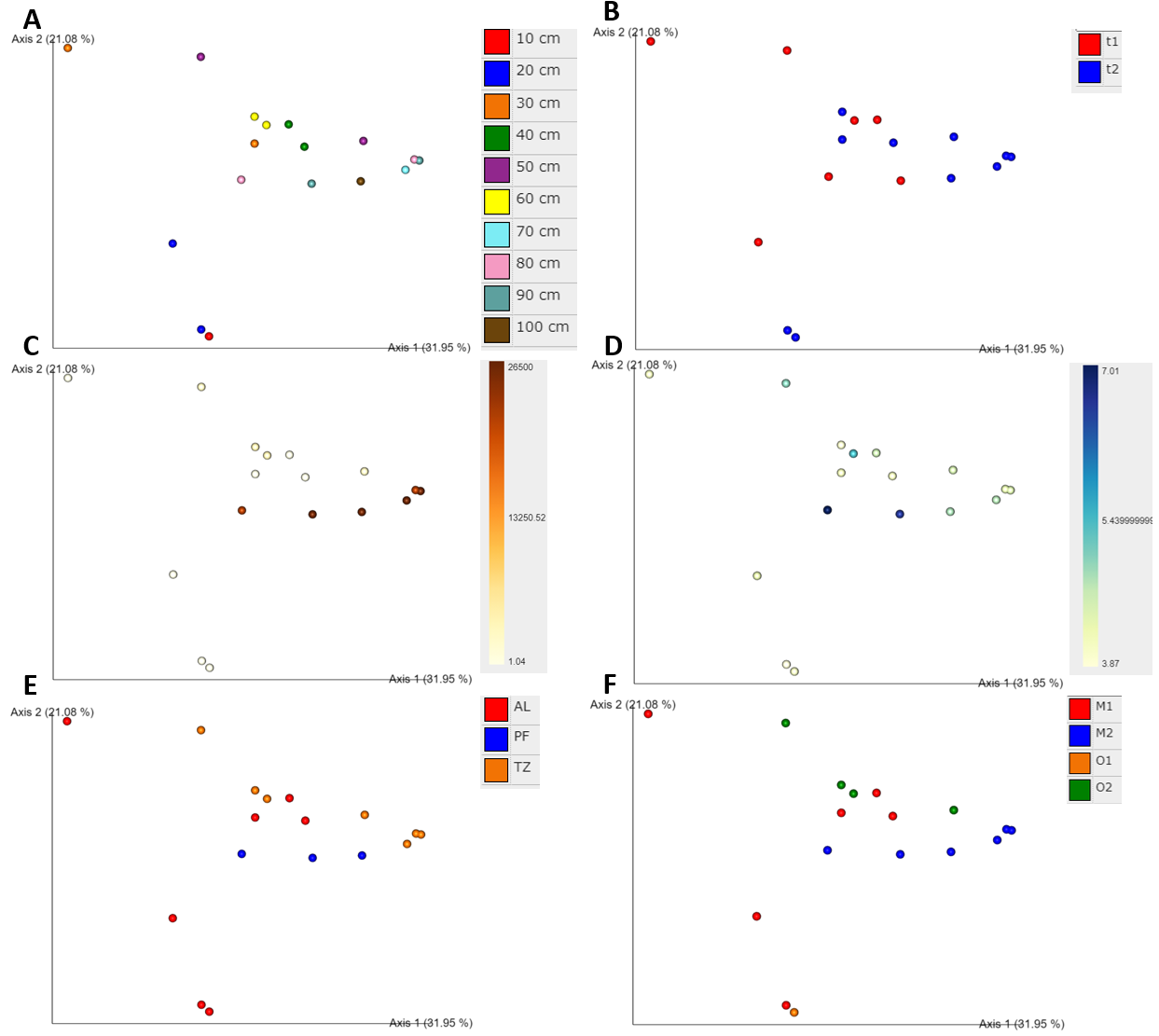


Supplementary Figure 5. Principal coordinate analysis (PCoA) results were visualized with QIIME2 Emperor plots for ITS Unifrac distances along the first two axes, each depicting the variability explained in %. Diamonds are used for 2019 and rings for 2020 measures. The graphs depict various soil parameter and their legend on the left, including depth (A), year (B), soil age ^14^C (C), pH (D), layer (E) and horizon (F). Abbreviations used herein include t1 for 2019, t2 for 2020, AL for active layer, PF for permafrost, TZ for transition zone, O1 for surface organic layer, M1 for top mineral layer, O2 for buried organic layer and M2 for ancient mineral layer, as defined in Tab. 1.


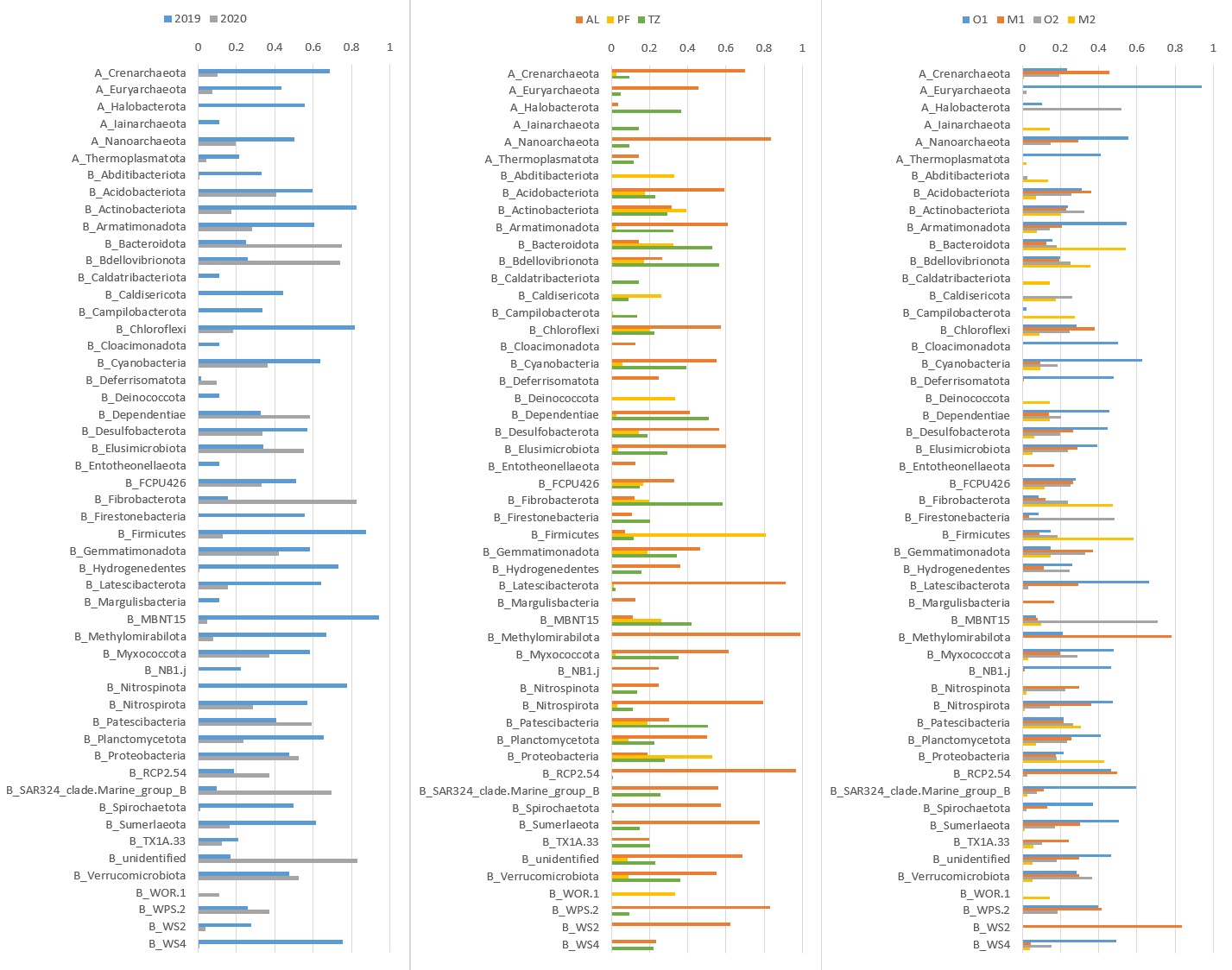


Supplementary Figure 6. Indval values obtained from the labdsv package within vegan indicate general significance per phylum in different environmental group contrasts (16S only). Left: contrasting both sampling ears 2019 and 2020. Middle: contrasting the layers AL, TZ and PF. Right: Contrasting the horizons O1, M1, O2, M2. Unidentified and ITS taxa were excluded.


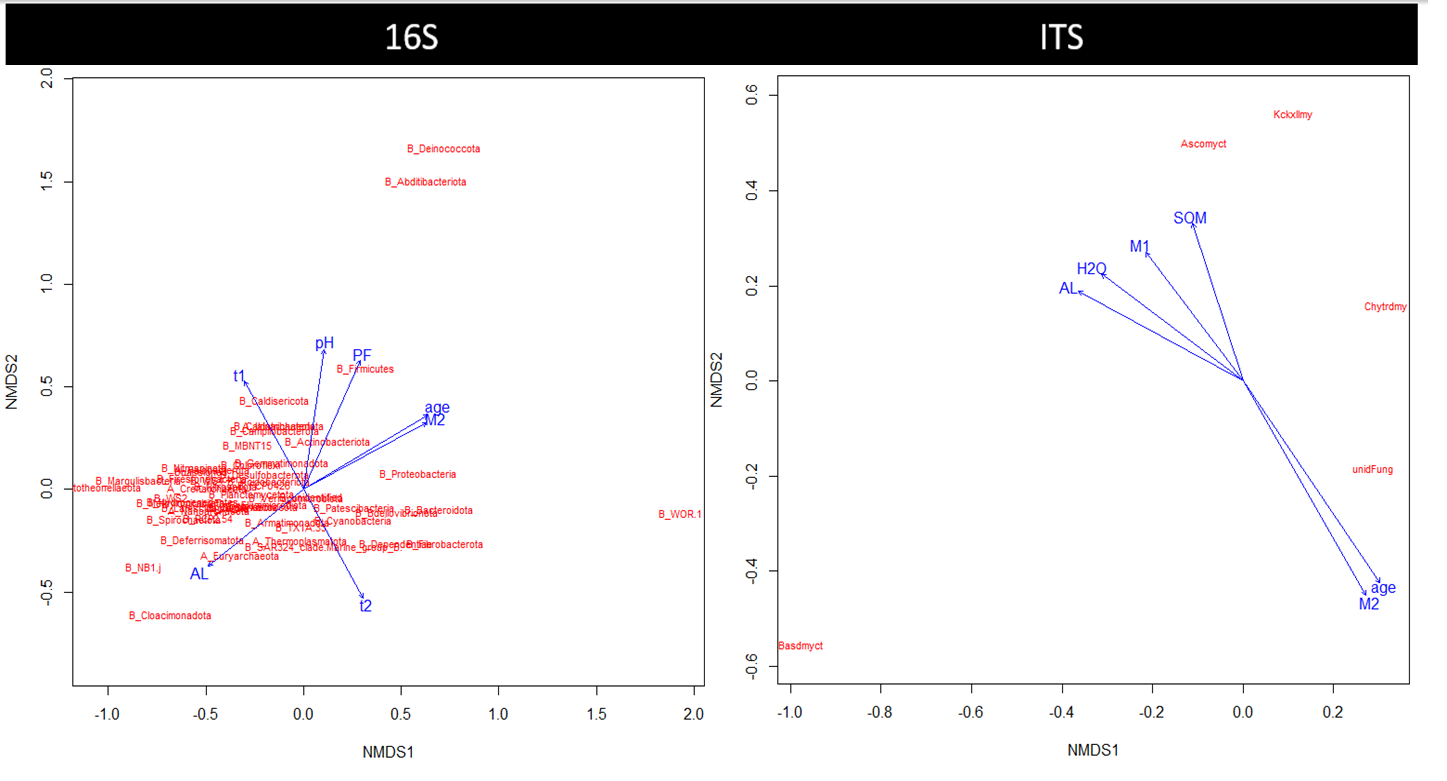


Supplementary Figure 7. Non-Metric Multidimensional (k=9) Scaling (NMDS) plots were performed on prokaryotic (16S) and fungal (ITS) Bray–Curtis (BC) dissimilarities. Phyla are indicated (red) with “B_” for bacterial and “A_” for archaeal taxa. Arrows (blue) indicate environmental parameters fitted onto the ordination, only including significant (P-value < 0.05) parameters, including the years 2019 (t1) and 2020 (t2), mineral top (M1) and buried (M2) horizons, layers active layer (AL) and permafrost (PF) and numeric environmental data radiocarbon dating (age), pH, H2O and SOM.
